# Olaparib combined with CDK12-IN-3 to promote genomic instability and cell death in ovarian cancer

**DOI:** 10.7150/ijbs.94568

**Published:** 2024-08-19

**Authors:** Jianqiang Liang, Xuan Zhou, Lin Yuan, Tian Chen, Yicong Wan, Yi Jiang, Huangyang Meng, Mengting Xu, Lin Zhang, Wenjun Cheng

**Affiliations:** Department of Gynecology, the First Affiliated Hospital of Nanjing Medical University, Nanjing 210029, Jiangsu, China.

**Keywords:** Olaparib, CDK12i, ovarian cancer, NHEJ, HR

## Abstract

Large-scale phase III clinical trials of Olaparib have revealed benefits for ovarian cancer patients with BRCA gene mutations or homologous recombination deficiency (HRD). However, fewer than 50% of ovarian cancer patients have both BRCA mutations and HRD. Therefore, improving the effect of Olaparib in HR-proficient patients is of great clinical value. Here, a combination strategy comprising Olaparib and CDK12-IN-3 effectively inhibited the growth of HR-proficient ovarian cancer in cell line, patient-derived organoid (PDO), and mouse xenograft models. Furthermore, the combination strategy induced severe DNA double-strand break (DSB) formation, increased NHEJ activity in the G2 phase, and reduced HR activity in cancer cells. Mechanistically, the combination treatment impaired Ku80 poly(ADP-ribosyl)ation (PARylation) and phosphorylation, resulting in PARP1-Ku80 complex dissociation. After dissociation, Ku80 occupancy at DSBs and the resulting Ku80-primed NHEJ activity were increased. Owing to Ku80-mediated DNA end protection, MRE11 and Rad51 foci formation was inhibited after the combination treatment, suggesting that this treatment suppressed HR activity. Intriguingly, the combination strategy expedited cGAS nuclear relocalization, further suppressing HR and, conversely, increasing genomic instability. Moreover, the inhibitory effect on cell survival persisted after drug withdrawal. These findings provide a rationale for the clinical application of CDK12-IN-3 in combination with Olaparib.

## Introduction

Maintenance therapy with poly (ADP-ribose) polymerase inhibitors (PARPi, such as Olaparib) is the most advanced regimen for ovarian cancer treatment and is used primarily for patients with BRCA1/2 mutations or homologous recombination deficiency (HRD), with its success corroborated by several phase III clinical trials^1^. Notably, this exclusive maintenance therapy has the ability to delay relapse^2^. However, only 30% of ovarian cancer patients have BRCA1/2 mutations, and fewer than 50% of patients with HRD also have BRCA1/2 mutations^3^. Thus, currently, the treatment options for the majority of ovarian cancer patients remain inadequate, and overcoming the specific genetic barrier to Olaparib application is an unmet need, as is the development of effective treatments for patients with HR proficiency; solving these problems is thus highly important.

The precise mechanism of Olaparib hinges on the concept of synthetic lethality. Specifically, Olaparib-induced DNA double-strand breaks (DSBs) cannot be repaired in cancer cells with HRD, resulting in cell death. Homologous recombination (HR) repair and nonhomologous end joining (NHEJ) are the two main pathways involved in repairing DSB damage, which is lethal to cells^4^. Notably, DNA end processing is pivotal for DSB repair initiation^5^ ; in this process, 53BP1 protects the DNA end from resection, which is executed by BRCA1, the MRE11-RAD50-NBS1 (MRN) complex, and CtIP, thus resulting in Ku70/80-mediated NHEJ^6^. Alternatively, when BRCA1 acts on DSBs, BRCA2-PALB2-RAD51 recruitment to replication protein A (RPA)-coated 5' overhangs in single-stranded DNA (ssDNA) predominantly activates HR repair^7^, ^8^. HR is generally considered to have high fidelity, whereas NHEJ is error prone. The choice between HR and NHEJ is intricately determined by the cell cycle phase, with HR occurring only during the mid‒late S phase and G2 phase, whereas NHEJ functions in the G1 phase and early S phase^9^. One fundamental mechanism controlling the balance between the two pathways is posttranslational modification, including phosphorylation/dephosphorylation, SUMOylation/deSUMOylation, and ubiquitylation/deubiquitylation^10^-^12^.

Recently, a combination strategy based on the DNA damage response (DDR) leading to the “BRCAness” phenotype, which increases the sensitivity of cancer cells to PARP inhibitors, has been widely used. AZD6738 (ceralasertib), an ATR inhibitor that is useful as a monotherapy or synergizes with Olaparib or carboplatin, results in robust tumor retardation through the modulation of ATR/ATM/DNA-PK signaling and HR repair^13^. Similarly, the combination of the CDK4/6 inhibitor palbociclib with Olaparib, which results in decreased HR activity in the G2 phase and irreparable DNA damage, inhibits the survival of triple-negative breast cancer (TNBC) cells^14^. Cyclin-dependent kinase 12 (CDK12), a member of the CDK family, first cooperates with Cyclin K to increase transcription elongation and genome stability^15^, ^16^. However, with the rapid development of multiple chemical compounds that target CDK12 and other CDKs, interesting questions have been raised: 1) As monotherapies, do these compounds have satisfactory or unsatisfactory effects? 2) Does a synergistic anticancer role exist when new synthetic compounds are combined with canonical PARP inhibitors (PARPi)? During the processing of our manuscript, Orhan E *et al.* discovered that the use of CDK12 inhibitors effectively enhances tumor sensitivity to PARPi in triple-negative breast cancer 17.

Here, we introduce a novel CDK12 inhibitor, CDK12-IN-3. We show that a combination strategy comprising CDK12-IN-3 and Olaparib enhances Ku80-mediated NHEJ activity via a noncanonical mechanism through fine-tuning of the PARP1-Ku80 complex, in which PARylation and phosphorylation of Ku80 are crucial for the PARP1-Ku80 interaction. Notably, G2 arrest occurs in ovarian cancer cells. Furthermore, HR activity is suppressed for DNA end protection and is also partially modulated through cGAS relocalization. Our findings in *in vitro* and *in vivo* models confirm this feasible and efficient regimen, which affects genome stability and has strong antitumor effects.

## Results

### Synthetic lethality of Olaparib and CDK12-IN-3 in HR-proficient ovarian cancer cells

PARP1 primes cells for single-strand break (SSB) repair first by interacting with DNA through its DNA binding domain (DBD) and then initiates DSB repair via its auto-modification domain (AD) and catalytic domain (CD) (Supplementary Fig. S1A). Olaparib (AZD2281), developed as a PARP1/2i, exploits the concept of synthetic lethality. CDK12-IN-3 (hereafter referred to as CDK12i), a new compound, is a highly selective CDK12 inhibitor that targets the CDK12 kinase domain (Supplementary Fig. S1B). Our cellular thermal shift assay (CETSA) demonstrated that the interaction of CDK12i with its target protein CDK12 markedly stabilizes the protein, significantly delaying its thermal denaturation as the temperature increases (Supplementary Fig. S1B). As SKOV3 and OVCAR3 are canonical HR-proficient ovarian cancer cell lines^18^, we selected them for our investigation. We initially attempted to use either CDK12i or CDK7-IN-1 (hereafter referred to as CDK7i) with Olaparib in SKOV3 and OVCAR3 cells; however, no synergistic effect was observed between CDK7i and Olaparib in either the dose response assay or time course assay, although both CDK7i and Olaparib exhibited a certain degree of anticancer effect per se (data not shown).

**Figure 1:**
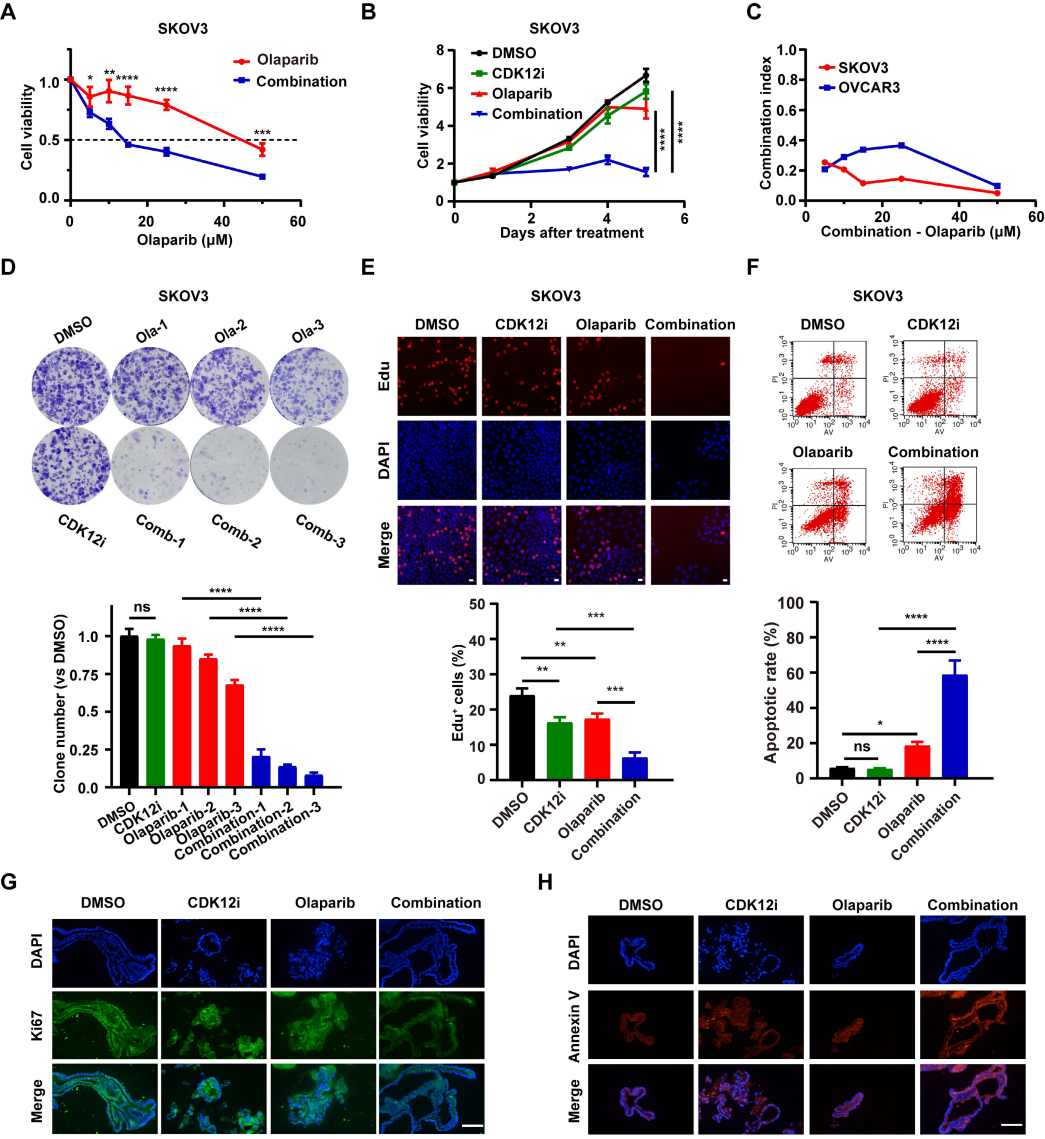
Synthetic lethality of Olaparib and CDK12-IN-3 in HR-proficient ovarian cancer cells. (A-B) A CCK-8 assay was used to evaluate the viability of SKOV3 cells after different treatments in dose-response (CDK12i: 50 nM; Olaparib: 0, 5, 10, 15, 25, or 50 μM) and time course (CDK12i: 50 nM; Olaparib: 10 μM) formats. n = 3 independent experiments. (C) Combination index values in SKOV3 and OVCAR3 cells. (D) Colony formation assay of SKOV3 cells subjected to different treatments. CDK12i: 15 nM, Ola-1: 0.5 μM, Ola-2: 1 μM, Ola-3: 2 μM. n = 3 independent experiments. (E) An EdU incorporation assay was used to assess the proliferation ability of SKOV3 cells (CDK12i: 50 nM, Olaparib: 10 μM, 2 days); scale bar: 20 μm. (F) Apoptosis was detected by flow cytometry in SKOV3 cells after various treatments for 48-72 h (CDK12i: 50 nM, Olaparib: 10 μM). n = 3 independent experiments. (G-H) IF staining for Ki67 and Annexin V in PDOs after treatment (CDK12i: 100 nM, Olaparib: 20 μM); scale bars: 75 μm.

Unexpectedly, however 50 nM CDK12i, which weakly impaired cancer cell viability, decreased cell viability when combined with Olaparib in various concentration combinations (Fig. 1A, Supplementary Fig. S1C and S1D). Even the cancer cells nearly completely eliminated under sustained treatment conditions (0-5 days); however, the effect in the negative control and single-drug groups was minimal (Fig. 1B and Supplementary Fig. S1E). The combination index (CI) is an indicator of the combinational effect of drugs and is calculated via the Chou‒Talalay method^19^. The combination index (CI) is an indicator of the combinational effect of drugs and is calculated via the Chou‒Talalay method^18^. The CI of the combination strategy tended to be zero, indicating the powerful synergistic cytotoxic activity of the combination treatment toward cancer cells (Fig. 1C). To further mimic the condition of maintenance therapy, a long-term colony formation assay was conducted. Indeed, the cytostatic effect was prominent in the presence of low concentrations of CDK12i and Olaparib and even peaked with an increased concentration of Olaparib (Fig. 1D and Supplementary Fig. S1F). Additionally, the results of the EdU incorporation assay revealed conspicuous growth retardation upon combination treatment but indiscernible suppression in the DMSO group and the single-drug groups (Fig. 1E and Supplementary Fig. S1G). Similarly, the apoptosis rate was estimated, and the results were consistent with those of the cell viability assay (Fig. 1F and Supplementary Fig. S1H). Importantly, in the preclinical organoid model, significant slowing of proliferation and acceleration of apoptosis were observed after the combination treatment, while these effects were limited after single-agent treatment (Fig. 1G-1H). Taken together, these findings shed light on the synthetic lethality of this drug combination at a reasonable concentration ratio, in contrast to the weak effects of either drug alone.

### The combination of CDK12i and Olaparib primes ovarian cancer cells for severe genomic instability and G2/M arrest

**Figure 2:**
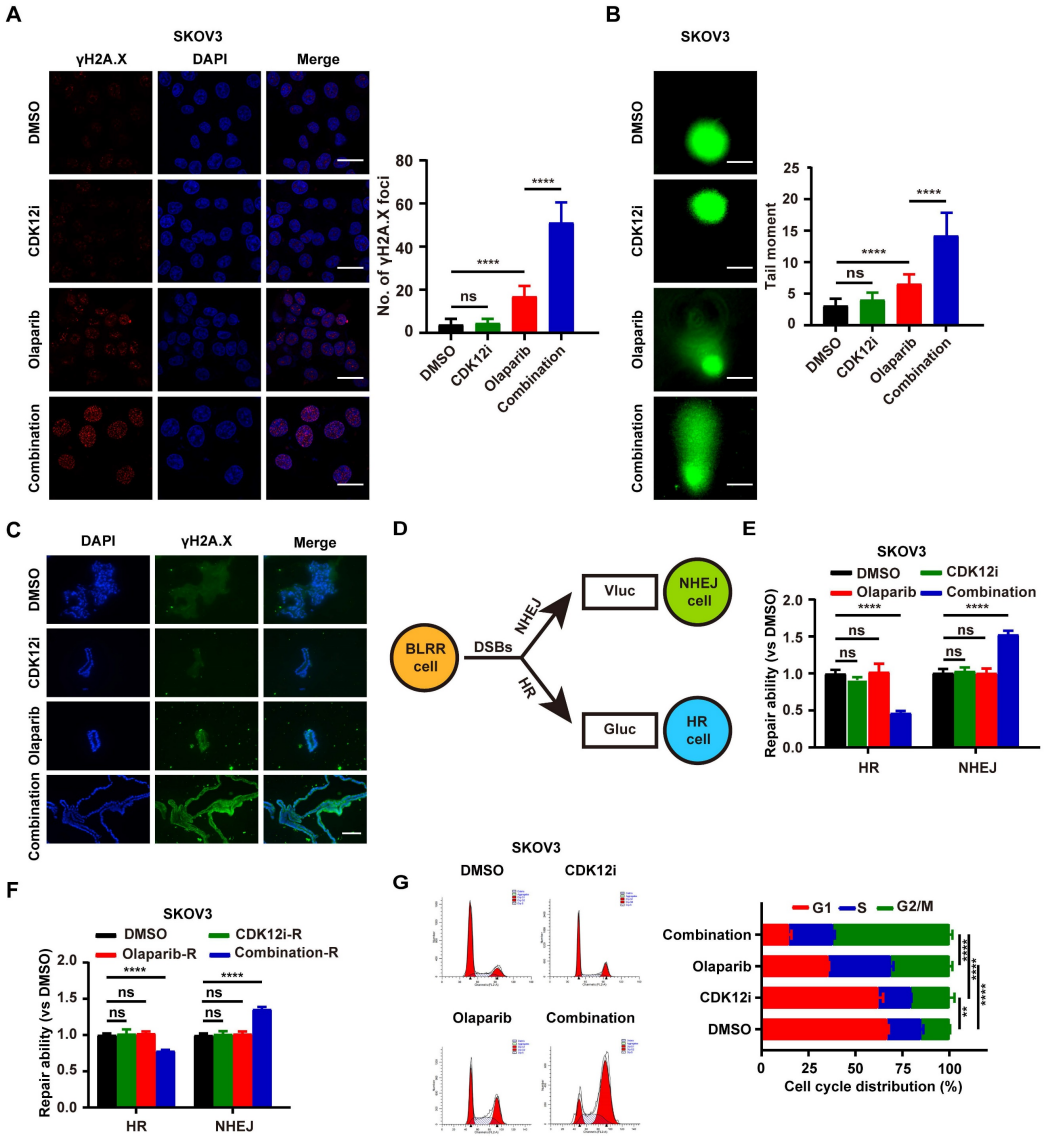
The combination of Olaparib and CDK12i primes ovarian cancer cells for severe genomic instability and G2/M arrest. (A) IF staining of γH2A.X foci to visualize DSBs in SKOV3 cells treated with drugs for 48 h (CDK12i: 50 nM, Olaparib: 10 μM); scale bar: 20 μm. (B) Comet assay showing changes in the genomic stability of SKOV3 cells after treatment for 48 h (CDK12i: 50 nM, Olaparib: 10 μM); scale bar: 10 μm. (C) IF staining of γH2A.X in PDOs after treatment (CDK12i: 100 nM, Olaparib: 20 μM); scale bar: 75 μm. (D) Diagram of the BLRR system. (E) The BLRR system demonstrated NHEJ activity and HR activity in SKOV3 cells after 48 h of treatment (CDK12i: 50 nM, Olaparib: 10 μM). n = 3 independent experiments. (F) After 48 h of treatment, SKOV3 cells were cultured for 48 h in fresh drug-free medium, after which the NHEJ activity and HR activity were tested via the BLRR system. n = 3 independent experiments. (G) Cell cycle distribution of SKOV3 cells treated with drugs for 48-72 h, as determined by flow cytometry (CDK12i: 50 nM; Olaparib: 10 μM). n = 3 independent experiments.

Endogenous DNA damage stress is induced via multiple cellular processes and may lead to cancer when it exceeds the capacity for high-fidelity DNA repair^20^. However, exogenous DNA damage also indicates cellular readiness and susceptibility to anticancer treatments such as cisplatin, radiation, or PARPi, which target DDR processes^21^. To explore the genomic integrity perturbation under drug combination, we monitored the γH2A.X foci, generally considered as a DSBs surrogate, to recapitulate the dynamic events. To explore the changes in genomic integrity caused by the drug combination, we monitored the formation of γH2A.X foci, which are generally considered a DSB marker, to evaluate dynamic events. After 48 h of treatment, CDK12i had little effect, similar to the effect of the control DMSO, whereas Olaparib caused low to moderate DSB formation. In contrast, the combination treatment resulted in an overt damage phenotype (Fig. 2A and Supplementary Fig. S2A). Consistent with the results of immunofluorescence (IF) staining, the alkaline comet assay demonstrated that genome integrity was severely impaired in the combination therapy group, possibly because of DDR deficiency or excessive DNA damage (Fig. 2B and Supplementary Fig. S2B). Moreover, this finding was verified by the genomic instability in patient-derived organoids (PDOs) upon the combination treatment (Fig. 2C).

Cellular DSB repair is performed mainly by the HR and NHEJ systems. To further investigate whether HR and NHEJ are disrupted, a novel multiplexed bioluminescent repair reporter (BLRR) system for tracking DSB repair pathway activity was used^22^. HR and NHEJ activity were simultaneously detected via secreted Gaussia luciferase (Gluc) and Vargula luciferase (Vluc), respectively (Fig. 2D). Surprisingly, NHEJ activity was hyperactivated under treatment with the combination strategy, with distinct impairment of HR. Notably, both NHEJ and HR were unimpeded in the other groups, possibly because the physiological DDR was sufficient to cope with the weak pressure induced by Olaparib or CDK12i alone (Fig. 2E and Supplementary Fig. S2C). In a treatment recovery assay, in the recovery period almost completely phenocopied HR/NHEJ activity in the treatment period in the combination group, even 48 h after recovery. In contrast, the HR/NHEJ activity in the CDK12i group was similar to that in the DMSO group and the Olaparib group (Fig. 2F and Supplementary Fig. S2D). Thus, these findings suggest that these two compounds cooperatively and disproportionately affect HR/NHEJ function relative to that under quiescent conditions or in the physiological controlled state, potentially resulting in persistent DSBs and failure to survive.

These phenotypes naturally raise the question of whether the cell cycle is altered, as the cell cycle is a central factor in the DNA damage signaling cascade and DNA end resection^23^. Surprisingly, the cells exhibited notable G2/M arrest under the combination therapy, suggesting the presence of catastrophic DSBs needing resolution. Interestingly, the cells in the Olaparib group exhibited minor G2/M arrest, which was also indicative of slight replication stress (Fig. 2G and Supplementary Fig. S2E). Thus, it is readily hypothesized that this drug combination might inappropriately hyperactivate the NHEJ pathway and inactivate the HR pathway in the G2 phase in response to DSBs induced by the drug combination itself, thus resulting in genome instability and consequent unavoidable cell death.

### Ku80 acts as the co-target of Olaparib and CDK12i

**Figure 3:**
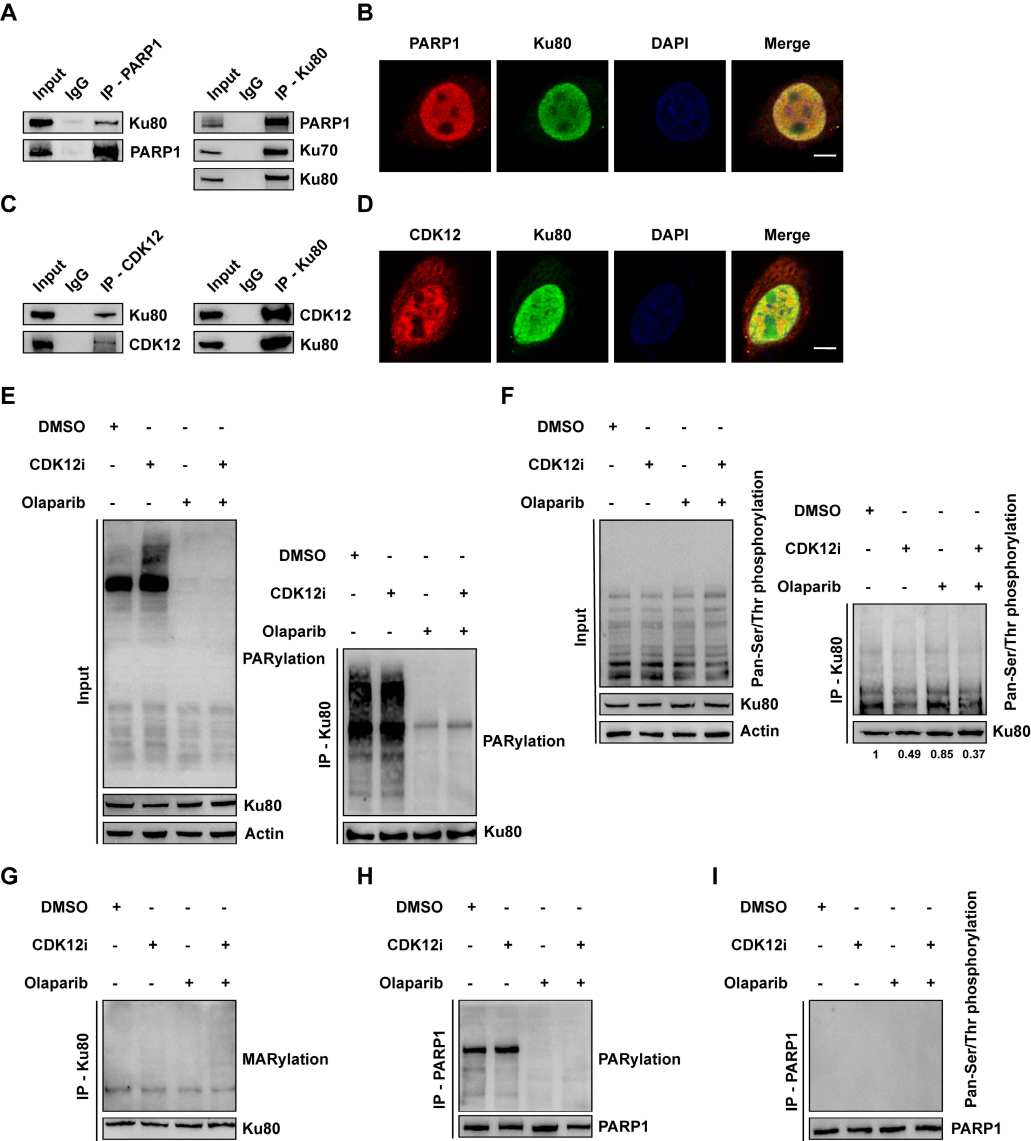
Ku80 acts as the co-target of Olaparib and CDK12i. (A) Co-IP assays were performed to verify the interaction between PARP1 and Ku80 in SKOV3 cells. (B) IF assays were used to visualize the colocalization of PARP1 and Ku80 in SKOV3 cells; scale bar: 5 μm. (C) The interaction between CDK12 and Ku80 in SKOV3 cells was verified via Co-IP assays. (D) IF assays were used to confirm the colocalization of CDK12 and Ku80 in SKOV3 cells; scale bar: 5 μm. (E) Ku80 Co-IP in SKOV3 cells subjected to different treatments (CDK12i: 50 nM, Olaparib: 10 μM, 2 days), followed by detection of PARylation via IB. (F) In SKOV3 cells subjected to different treatments (CDK12i: 50 nM, Olaparib: 10 μM, 2 days), Ku80 Co-IP was performed, and pan-Ser/Thr phosphorylation was then detected via IB. (G) Ku80 Co-IP was performed in SKOV3 cells subjected to different treatments (CDK12i: 50 nM, Olaparib: 10 μM, 2 days), and MARylation was then detected via IB. (H) PARP1 Co-IP was performed in SKOV3 cells subjected to different treatments (CDK12i: 50 nM, Olaparib: 10 μM, 2 days), and PARylation was then detected via IB. (I) PARP1 Co-IP was performed in SKOV3 cell lysates (CDK12i: 50 nM, Olaparib: 10 μM, 2 days), and pan-Ser/Thr phosphorylation was then detected via IB.

Although CDK12 functions as a negative mediator of HR, CDK12i had only a weak effect on HR activity (Fig. 2D-2E and Supplementary Fig. S2C-S2D). Accordingly, to determine whether Olaparib and CDK12i share and coregulate the same target, which could explain their synthetic lethality, we conducted mass spectrometry (MS) analysis. Considering that the targets of Olaparib are PARP1 and PARP2 and that the main executor is PARP1, we performed coimmunoprecipitation (Co-IP) for PARP1, followed by MS. Unexpectedly, we identified Ku80 (encoded by XRCC5) (Supplementary Fig. S3A), which binds to blunt DNA ends and forms a complex with Ku70 (encoded by XRCC6) and the DNA-dependent protein kinase catalytic subunit (DNA-PKcs; termed the trimeric DNA-PK holoenzyme), a main component of the NHEJ pathway^24^. Indeed, Co-IP of PARP1 revealed a close interaction between PARP1 and Ku80. Consistent with this finding, PARP1 was also enriched in the Ku80 immunoprecipitate, and Ku70 was also identified as a bona fide interacting partner (Fig. 3A). IF assays further verified the presence of the PARP1-Ku80 complex under physiological conditions (Fig. 3B and Supplementary Fig. S3B). Notably, the relationship between CDK12 and Ku80 was confirmed to be a direct physical association and similar to that between PARP and Ku80 (Fig. 3C). The IF assay results confirmed this physiological interaction (Fig. 3D and Supplementary Fig. S3C).

Given the catalytic action of PARP1 and CDK12, we first evaluated the total poly(ADP-ribosyl)ation (PARylation) and phosphorylation levels under treatment. First, we found that the expression of CDK12, PARP1 or Ku80 did not change, precluding the interference of the drugs with protein expression (Supplementary Fig. S3D). Both Olaparib and the combination treatment reduced the total PARylation level, whereas a sharp increase in total PARylation was observed under CDK12i treatment (Fig. 3E). This effect might be limited to several targets, as neither CDK12i treatment nor the combination therapy had little effect on the total phosphorylation level (Fig. 3F). Next, we explored whether the chemical modifications of Ku80 were affected by both Olaparib and CDK12i. To this end, we conducted a Co-IP assay for Ku80 followed by immunoblotting to evaluate its PARylation and phosphorylation. Indeed, Olaparib and the combination treatment distinctly inhibited the PARylation of Ku80, whereas CDK12i had no effect on Ku80 PARylation (Fig. 3E). Similarly, CDK12i treatment decreased the phosphorylation of Ku80, and the combination treatment phenocopied this reduction. Notably, no difference in phosphorylation was observed between the Olaparib and DMSO groups (Fig. 3F). Thus, Olaparib and CDK12i directly inhibited Ku80 PARylation and phosphorylation, respectively, whereas no crossover effect of Olaparib on phosphorylation or of CDK12i on PARylation was detected.

Mono(ADP-ribosyl)ation (MARylation), which is catalyzed by PARP family members, including PARPs 3, 4, 6-12, and 14-16, and removed by diverse ADPR hydrolases, is pivotal for DDR processes^25^. Thus, it is tempting to speculate that the MARylation of Ku80 is disrupted upon combination therapy. In response to DSBs, total MARylation was increased after the combination treatment (Supplementary Fig. S3E). Disappointingly, no overt alteration was detected in Ku80 in either single-drug group or in the combination group (Fig. 3G), indicating that Ku80 MARylation did not occur. In addition, we conducted Co-IP for PARP1 and examined whether its PARylation and phosphorylation were decreased. Consistent with the findings of a previous report^26^, the PARylation of PARP1 was severely impaired upon treatment with Olaparib or the combination, whereas CDK12i did not affect PARylation (Fig. 3H and Supplementary Fig. S3F). Unexpectedly, it was difficult to detect the phosphorylation of PARP1, even in the DMSO control group (Fig. 3I and Supplementary Fig. S3G), implying that PARP1 is not the target of CDK12 and that the physiological role of PARP1 phosphorylation is of marginal importance. Thus, these findings indicate that Olaparib cooperates with CDK12i, directly resulting in dePARylation and dephosphorylation of Ku80, which are unrelated to MARylation.

### The combination therapy aberrantly activates NHEJ via modification-dependent dissociation of the PARP1-Ku80 complex

**Figure 4:**
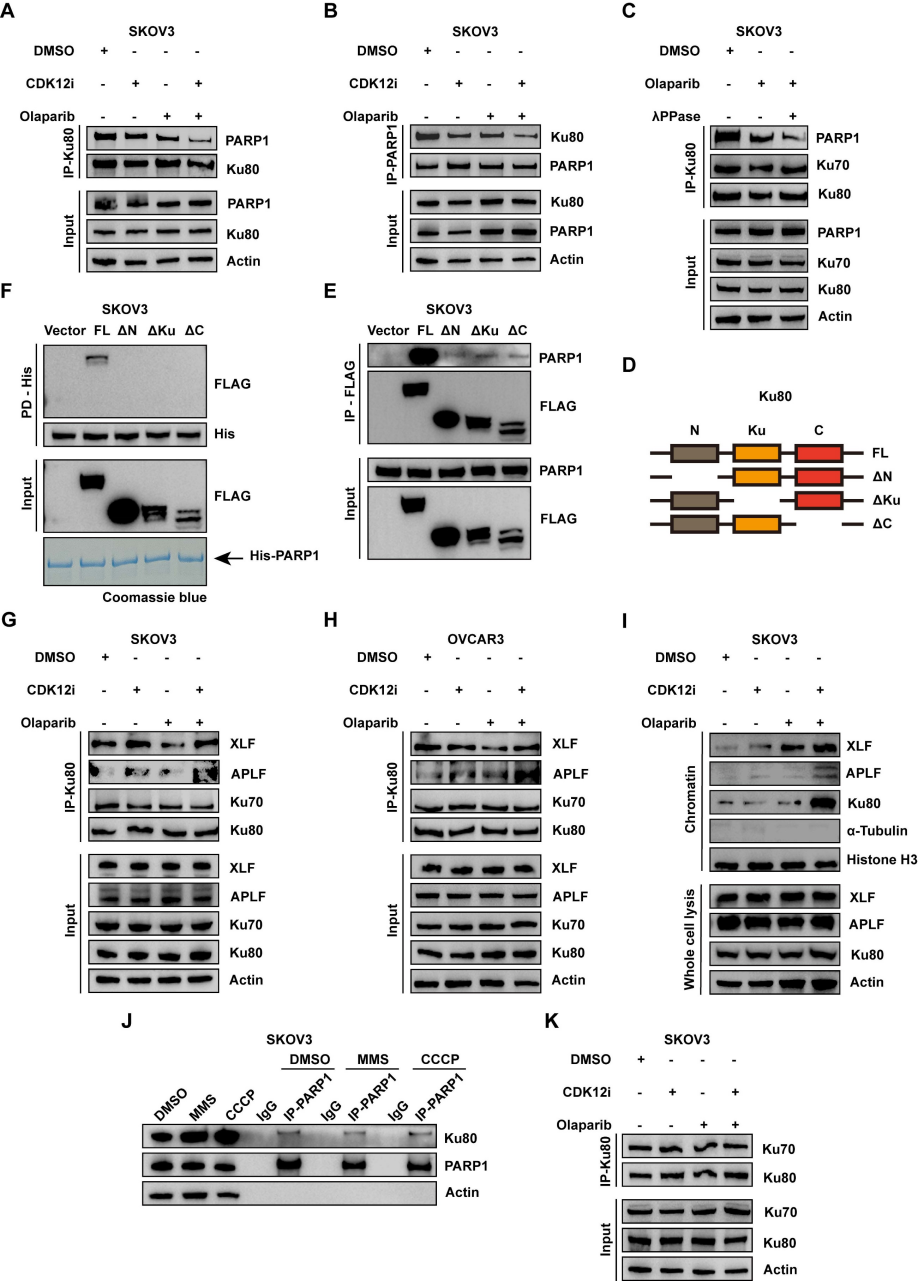
Combination therapy aberrantly activates NHEJ via modification-dependent dissociation of the PARP1-Ku80 complex. (A-B) Co-IP of Ku80 and PARP1 in SKOV3 cells subjected to various treatments revealed the stability of the PARP-Ku80 complex (CDK12i: 50 nM, Olaparib: 10 μM, 2 days). (C) After treatment with DMSO or Olaparib (10 μM, 2 days), λPPase was added to Olaparib-treated SKOV3 cell lysates. Then, Ku80 Co-IP was performed, followed by IB. (D) Mapping of full-length Ku80 and its truncated variants. (D) The mapping of full-length Ku80 and truncated variants. (E) After HEK293T cells were transfected with a series of 3×FLAG-tagged Ku80 mutants for 48 h, as depicted in (D), FLAG Co-IP was conducted to identify the interaction domain of Ku80 with PARP1. (F) *In vitro* pulldown assay in which recombinant 6×His-PARP1 protein was mixed with HEK293T cell lysates transfected with 3×FLAG-Ku80 truncation mutants. (G-H) Ku80 Co-IP followed by IB was used to investigate the Ku80-XLF-APLF complex under different treatment conditions in SKOV3 and OVCAR3 cells (CDK12i: 50 nM, Olaparib: 10 μM, 2 days). (I) A chromatin fractionation assay was used to confirm the increase in Ku80-mediated NHEJ activity in SKOV3 cells (CDK12i: 50 nM, Olaparib: 10 μM, 2 days). (J) Under standard culture conditions, SKOV3 cells were treated with DMSO for 1 h, 0.01% MMS for 1 h, or 80 μM CCCP for 2 h, and PARP1 Co-IP was then conducted to evaluate the stability of the PARP1-Ku80 complex. (K) Ku80 Co-IP was used to investigate the stability of the Ku70/80 complex in SKOV3 cells after different treatments (CDK12i: 50 nM, Olaparib: 10 μM, 2 days).

The biological function of concurrent PARylation and phosphorylation of the same target is poorly understood. A previous study revealed that PARP1 and the Ku70/80 complex are simultaneously recruited to DSBs earlier than other DSB sensors are; the Ku complex occupies DNA lesions in the G1 phase, whereas PARP1 evicts the Ku complex through its enzymatic activity in the S/G2 phase^27^. Considering that potent G2/M arrest occurs upon the combination treatment (Fig. 2F and Supplementary Fig. S2E), accompanied by numerous DSBs (Fig. 2A-2B and Supplementary Fig. S2A-S2B), we hypothesized that the PARP1-Ku80 interaction would first be enhanced and that consequently, Ku80 would be lost because of the evicting activity of PARP1 at DSBs. Considering the pivotal role of the PARP1-Ku80 complex in the DDR and the choice of repair pathway, we investigated whether the PARP1-Ku80 complex was dissociated or markedly controlled by the PARylation and phosphorylation of Ku80. The results of the Co-IP assay demonstrated minor dissociation when cells were treated with either Olaparib or CDK12i, whereas apparent dissociation occurred with the combination treatment (Fig. 4A). Reciprocally, the results of the PARP1 Co-IP assay confirmed the corresponding relationship (Fig. 4B). To further explore the biological role of Ku80 PARylation and phosphorylation in PARP1-Ku80 complex dissociation, we applied λPPase (a protein phosphatase that dephosphorylates multiple amino acid residues) to the cell lysate. As expected, the PARP1-Ku80 complex was severely disrupted by simultaneous treatment with Olaparib and λPPase (Fig. 4C). In the combination treatment group, we utilized PARG inhibitors (PARGi) to enhance the PARylation of Ku80. Subsequent co-immunoprecipitation experiments revealed that the PARP1-Ku80 complex was partially restored following the addition of PARGi (Fig. S4A). The result indicates that the combination drug regimen induced the dissociation of the PARP1-Ku80 complex, underscoring the critical role of Ku80 PARylation and phosphorylation in maintaining the stability of this complex.

Next, to map the domain involved in the interaction of Ku80 with PARP1, we constructed a series of Ku80 truncations fused to a 3×Flag tag at the C-terminus (Fig. 4D). Surprisingly, only FL interacted with PARP1, with ΔN, ΔKu, and ΔC capturing almost no PARP1 (Fig. 4E). Moreover, the *in vitro* pulldown assays confirmed that only FL directly associated with 6×His-PARP1 (Fig. 4F). Thus, we speculated that the PARylation sites and phosphorylation sites regulated by PARP1 and CDK12 on Ku80 were distributed from the N-terminus to the C-terminus and that the combined modification of most of these sites, rather than the modification of a single site, were crucial to the PARP1-Ku80 complex. Specifically, the phosphorylation sites in Ku80 were distributed from T39 to T715; however, no information about the PARylation sites in Ku80 was available^28^. Further investigations might reveal the specific PARylation sites in Ku80, with the potential identification of two modification-dependent bridges between PARP1 and Ku80.

Given the impaired competition between PARP1 and Ku80 in the G2 phase and the increased NHEJ activity, it is tempting to speculate that Ku80-mediated NHEJ is extremely active in the G2 phase. A previous study reported that PP2A (a main Ser/Thr phosphatase) directly dephosphorylates Ku and DNA-PKcs, thus increasing NHEJ activity via the formation of a functional Ku/DNA-PKcs complex^29^, indicating the critical role of posttranslational modifications in the modulation of NHEJ activity. Thus, we performed a Co-IP assay, which revealed prominent assembly of the Ku80/APLF/XLF complex under the combination treatment (Fig. 4G-4H). In particular, XLF and APLF are two initial responsive anchors recruited by Ku80, partially governing the NHEJ pathway^30^. To further characterize the biological significance of the PARylation- and phosphorylation-dependent PARP1-Ku80 complex in Ku80-mediated NHEJ, a chromatin fractionation assay was employed. Under the combination treatment, Ku80 was overtly enriched in chromatin, whereas no perceivable change in its abundance was detected in the single-drug groups or the DMSO group (Fig. 4I and Supplementary Fig. S4C). In line with this finding, the enrichment of APLF and XLF exhibited the same trend as that of Ku80 (Fig. 4I and Supplementary Fig. S4C). To delineate the ability for damage processing in the presence of MMS (a DNA-damaging agent) in the treatment groups, we performed costaining for Ku80 and γH2A.X at 0 h and 2 h after transient MMS treatment. At 0 h of recovery, the abundance of Ku80 was drastically increased in the combination group and remained increased at 2 h of recovery (Supplementary Fig. S4B). With respect to genome stability, more severe and persistent DSBs were observed in the combination group than in the other groups, but the presence and severity of DSBs was restored to the baseline in the other groups at 2 h of recovery (Supplementary Fig. S4B). Thus, the dePARylation and dephosphorylation of Ku80 induced by Olaparib and CDK12i resulted in dissociation of the PARP1-Ku80 complex, thus aberrantly increasing Ku80-mediated NHEJ activity in the G2 phase and resulting in genome instability.

As mentioned above, the PARP1-Ku80 complex is crucial for maintaining genomic stability. Notably, the PARP1-Ku80 complex was stable when noncancerous cells were exposed to some DNA-damaging agents, as determined via Co-IP assays (Fig. 4J and Supplementary Fig. S4D). We further explored whether the Ku70/80 complex was disrupted by the combination strategy. Both the Ku80 Co-IP and the Ku70 Co-IP showed that the complex remained in equilibrium in all four groups (Fig. 4K and Supplementary Fig. S4E). In addition, we sought to determine whether the formation of 53BP1 foci differed under the various treatment conditions. However, there was no difference among the groups (Supplementary Fig. S4F). Thus, the combination strategy-mediated activation of NHEJ in the G2 phase was independent of 53BP1 recruitment but dependent on Ku80 occupancy of DSBs.

### Nuclear relocalization of cyclic GMP-AMP synthase (cGAS) under the combination treatment inhibits HR activity

**Figure 5:**
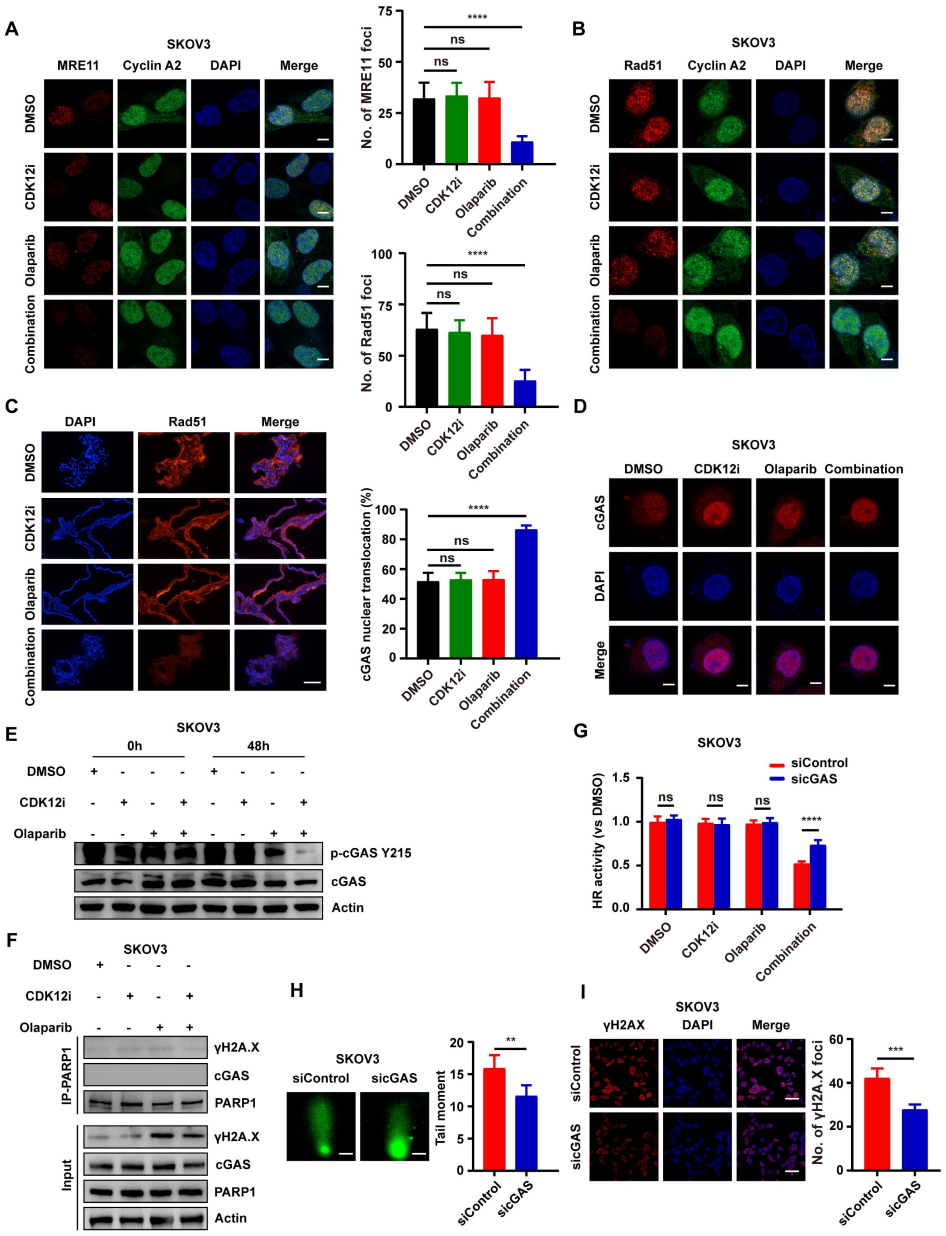
Nuclear relocalization of cGAS under the combination treatment inhibits HR activity. (A-B) Visualization of MRE11 foci and Rad51 foci via IF staining in SKOV3 cells subjected to different treatments (CDK12i: 50 nM, Olaparib: 10 μM) for 48 h followed by transient 0.01% MMS treatment; scale bar: 5 μm. (C) IF staining of Rad51 in PDOs after treatment (CDK12i: 100 nM, Olaparib: 20 μM); scale bar: 75 μm. (D) IF was used to visualize the cGAS distribution in SKOV3 cells (CDK12i: 50 nM, Olaparib: 10 μM, 2 days); scale bar: 5 μm. (E) cGAS phosphorylation at Y215 in SKOV3 cells was detected via IB using a specific antibody (CDK12i: 50 nM, Olaparib: 10 μM, 2 days). (F) A Co-IP assay was performed to investigate whether PARP1 interacts with cGAS in SKOV3 cells (CDK12i: 50 nM, Olaparib: 10 μM, 2 days). (G) The BLRR system was used to evaluate the role of cGAS in HR in SKOV3 cells (CDK12i: 50 nM, Olaparib: 10 μM, 2 days). n = 3 independent experiments. (H-I) An alkaline comet assay (scale bar: 10 μm) and IF staining (scale bar: 20 μm) were used to investigate the role of cGAS in contributing to genomic stability in SKOV3 cells (CDK12i: 50 nM, Olaparib: 10 μM, 2 days).

As the Ku complex can protect blunt DNA ends from resection, the MRN complex might be unable to perform DNA end resection under the combination treatment. To distinguish cells in the S/G2 phase from cells in the G1 phase, cyclin A was used. As expected, after transient MMS addition, the number of MRE11 foci was dramatically reduced in the combination group but was unaffected in the single-drug groups (Fig. 5A and Supplementary Fig. S5A). Another core downstream executor, Rad51, which has recombinase activity, searches for and invades homologous DNA duplexes, thus forming a displacement loop (D-loop), which further accelerates HR repair^31^. Indeed, the pattern of changes in Rad51 foci was similar to that observed for MRE11 foci (Fig. 5B-5C and Supplementary Fig. S5B), suggesting the impairment of HR repair.

DNA DSBs can contribute to the release of free DNA fragments into the cytosol. Importantly, cGAS induces an interferon-β-mediated immune response through its sensing of cytosolic DNA^32^. However, an increasing number of studies focused on the atypical function of cGAS have revealed that the nuclear localization of cGAS attenuates HR activity, thus increasing genome instability^33^, ^34^. Thus, we hypothesized that cGAS contributes to this. Surprisingly, nuclear cGAS localization was increased under the combination treatment but was not affected by either single-drug treatment or the negative control treatment (Fig. 5D and Supplementary Fig. S6A). Consistent with the observation that the phosphorylation of cGAS at Y215 controls its nuclear transport^33^, cGAS Y215 phosphorylation was nearly completely abolished after 48 h of the combination treatment (Fig. 5E and Supplementary Fig. S6B), and this large decrease was consistent with the nuclear relocalization of cGAS.

Liu and colleagues elaborated cGAS impeded HR activity via cGAS-PARP1 interaction, which further impaired PARP1-Timeless formation^33^. Nevertheless, the cGAS-PARP1 interaction was not apparent in either the single-drug groups or the combination group, nor was the cGAS-γH2A.X interaction (Fig. 5F and Supplementary Fig. S6C). IF assays indicated weak co-localization between PARP1 and cGAS again (Supplementary Fig. S6D-S6E). By downregulating cGAS, there was a notable attenuation in DNA damage within the co-administered group, while Ku80 exhibited no discernible alterations (Supplementary Fig. S6L). In order to delve deeper into whether cGAS impacts the functionality of the PARP1-Ku80 complex, co-immunoprecipitation (COIP) experiment was conducted. The finding indicates that the downregulation of cGAS within the co-administered group does not perturb the outcome of PARP1-Ku80 complex stability disruption (Supplementary Fig. S6M). These findings suggest that cGAS does not exert an influence on Ku80 and the PARP1-Ku80 complex.

To better elucidate the role of cGAS nuclear localization in ovarian cancer cells, the BLRR system was used. HR activity was partially restored after cGAS knockdown under the combination treatment—this effect was unique to this group and not evident in the other groups; in contrast, NHEJ activity was unchanged (Fig. 5G and Supplementary Fig. S6F-S6I). Similarly, cGAS knockdown partially attenuated DNA damage after the combination treatment, as shown by the alkaline comet and IF assays (Fig. 5H-5I and Supplementary Fig. S6J-S6K). Thus, from another perspective, cGAS nuclear relocalization stimulated by the combination treatment inhibited HR activity and caused genomic instability.

### The patterns of several biomarkers reflecting cell fate

**Figure 6:**
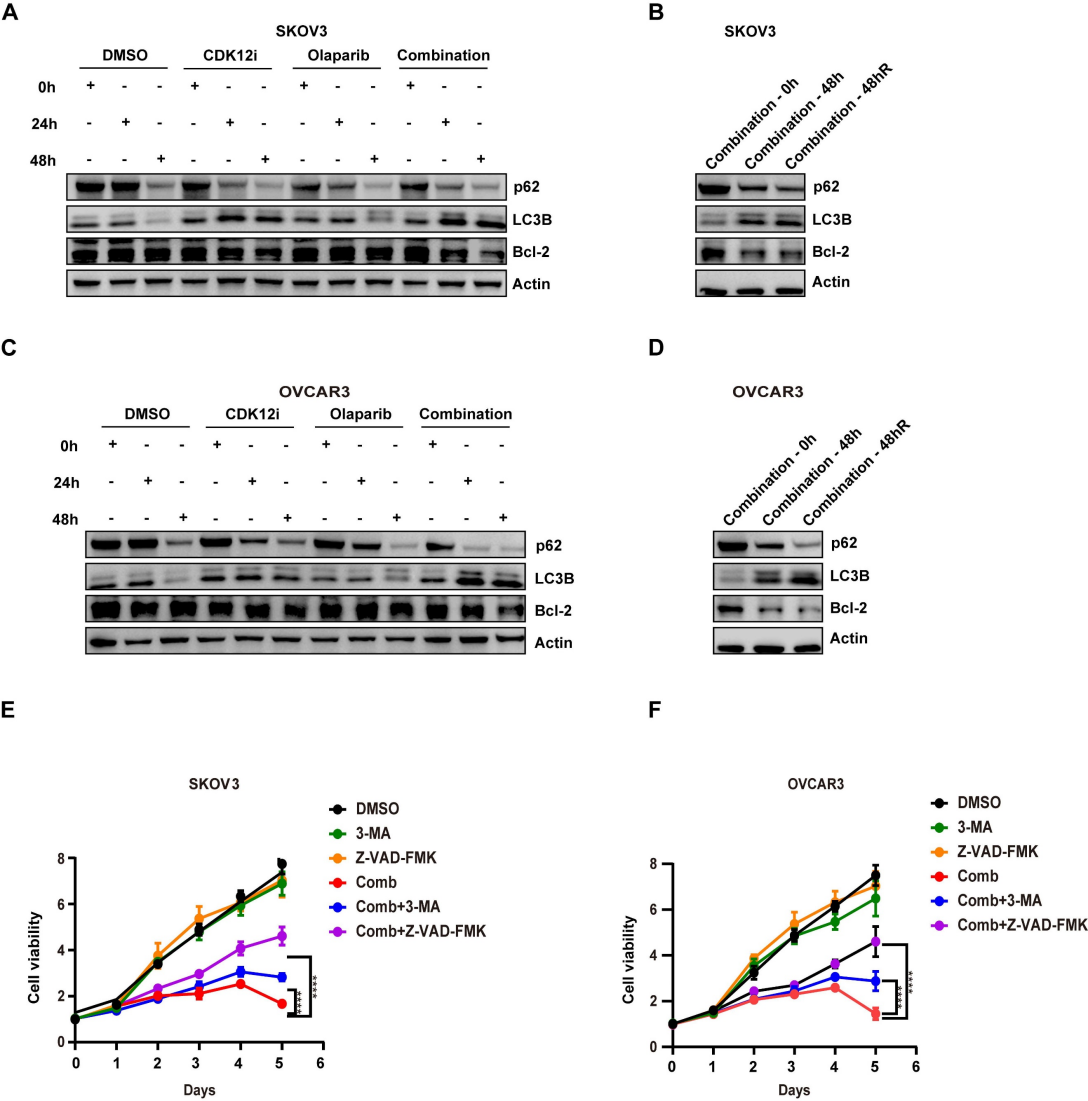
Expression patterns of several biomarkers indicating cell fate. (A-B) The expression of the antiapoptotic marker bcl-2, as well as the autophagy markers LC3B and p62, in SKOV3 cells was evaluated via IB. (C-D) The expression of the antiapoptotic marker bcl-2, as well as the autophagy markers LC3B and p62, in OVCAR3 cells was evaluated via IB. (E-F) A CCK-8 assay was used to evaluate the viability of SKOV3 and OVCAR3 cells after different treatments in dose-response (3-MA: 2.5 mM; Z-VAD-FMK: 15 μM; combination: CDK12i: 50 nM; Olaparib: 50 μM) and time course formats. n = 3 independent experiments.

Evidence indicates that the presence of toxic DSBs exceeding a threshold is a potent activator of apoptosis, especially in cells with failure of DSB repair, along with collapsed replication forks^35^. In contrast to the conditions of continuous administration of agents in the clinic and in preclinical models, in our study, the medium was replaced with fresh, drug-free medium after 48 h of treatment with either single agent or the combination, and the cells were cultured for an additional 48 h. Surprisingly, even after time for recovery, the expression of Bcl-2, the main marker of apoptosis, was similar to that observed under the combination treatment (Fig. 6A-6D). This reduction was not observed in the single-agent groups, probably because the repair factors were effective (Fig. 6A and 6C).

Accumulating evidence indicates that the presence of DSBs activates autophagy via ATM-mediated signaling or some posttranslational modifications, which in turn affect genome stability^36^. After short-term (24 h) combination treatment, autophagy was sharply increased, with an even more prominent increase at 48 h (Fig. 6A-6D). This pattern was indicative of the presence of severe and persistent DSBs, although CDK12i treatment resulted in phenocopying. Similar to apoptosis, autophagy was activated after release (Fig. 6B and 6D). One explanation for this dual activation is the crosstalk between autophagy and apoptosis. On the other hand, in response to DNA damage, when cytotoxic autophagy becomes cytotoxic, genomic integrity is destroyed and cell death occurs. However, in the combination treatment group when apoptosis or autophagy was suppressed, a substantial reduction in the tumoricidal effects of the combined treatment was evident upon apoptosis inhibition. This observation suggests that the robust antitumor effect of the Olaparib/CDK12-IN-3 combination stems primarily from the activation of apoptosis via the induction of toxic DSBs exceeding a critical threshold rather than from the activation of autophagy (Fig. 6E-6F).

As mentioned above, dramatic G2 arrest was observed in the combination group. Cyclin A was strongly downregulated, accompanied by a decrease in CDK2 expression (Supplementary Fig. S7A-S7D). Previous research has shown that Cyclin A/CDK2 contribute to the phosphorylation of CtIP and the resulting assembly of the MRN-CtIP-BRCA1 end resection complex^37^, consistent with our results. Unexpectedly, the expression of CDK4/6, which were previously reported to participate in a synergistic relationship with Olaparib in TNBC^14^, was also inhibited (Supplementary Fig. S7A-S7D), implying the role of a more intricate network between the cell cycle and the DDR in determining cell fate. After recovery, the expression patterns of cell cycle checkpoints were not altered in the combination group (Supplementary Fig. S7B and S7D), suggesting persistent DNA damage and G2 phase arrest.

Intriguingly, high-dose chemotherapy or radiotherapy predominantly induce apoptosis, whereas low-dose treatment triggers senescence^38^. β-Gal staining revealed the senescence-promoting role of Olaparib in ovarian cancer cells; however, apoptosis was increased and sustained when Olaparib was combined with CDK12i (Supplementary Fig. S7E‒S7H). This pattern may indicate a transition from senescence to apoptosis, consistent with the “one-two punch” concept^39^.

### The tumor retardation effect of combination therapy *in vivo*

**Figure 7:**
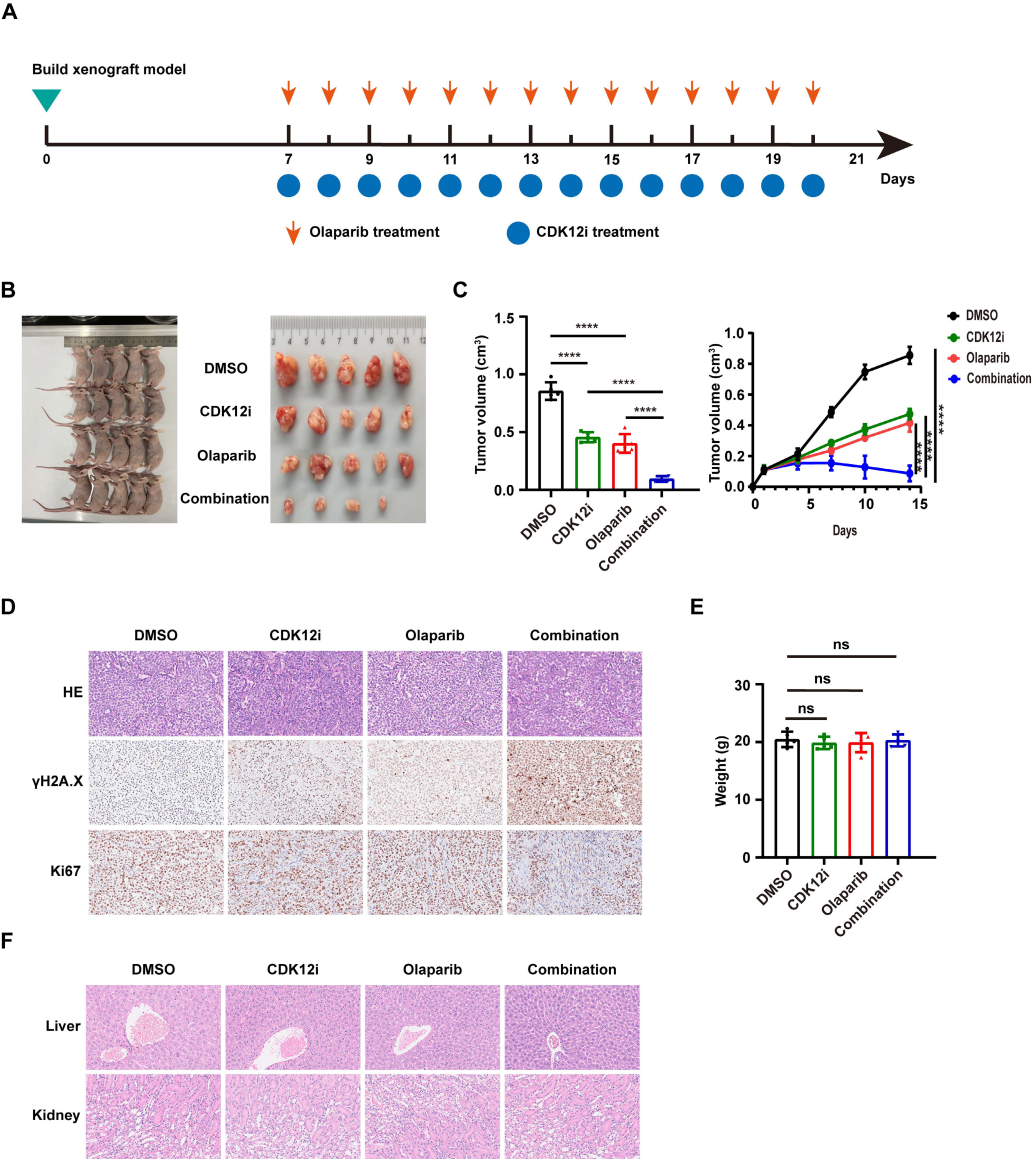
Tumor growth inhibitory effect of the combination therapy *in vivo*. (A) Flowchart of the animal experiment. (B-C) The results of the animal experiment demonstrated the notable tumor inhibitory effect of the combination treatment. n = 3 biological replicates. (D) IHC and HE staining were performed to evaluate the expression of the proliferation marker Ki67 and the DSB marker γH2A.X in xenograft tumor tissues. (E) Body weights of the mice after 21 days of treatment. (F) HE staining was used to assess the side effects of the treatment on the structure of the liver and kidney.

To further verify the robust antitumor effect of the combination therapy *in vivo*, we established a xenograft mouse model followed by 14 days of drug administration. Similar to the effects observed *in vitro*, Olaparib or CDK12i alone slightly delayed tumor progression, whereas the combination therapy substantially retarded tumor growth (Fig. 7A-7C). Through immunohistochemical (IHC) staining, it was apparent that the γH2A.X intensity in the combination group was much greater than that in either single agent group (Fig. 7D). Analysis of another proliferation marker, Ki-67, further confirmed the antitumor effect of the combination therapy (Fig. 7D).

To evaluate the side effects of the combination regimen, we measured the weights of the nude mice in each group. No significant difference was observed among the groups (Fig. 7E). To characterize the potential toxicity more accurately, the morphological structure of the liver and kidney were evaluated via hematoxylin‒eosin (HE) staining. The integrity of the microstructure was satisfactorily maintained in the single-agent and combination treatment groups (Fig. 7F). These findings suggest the favorable tolerance and minor side effects of the combination treatment *in vivo*.

## Discussion

In previous studies, several PARPi-containing combination strategies have been discussed and verified; these strategies include PARPi combined with antiangiogenic agents, PI3K/Akt/mTOR inhibitors, and HDAC inhibitors^40^-^42^. Through these combinations, cancer cells, though HR-proficient under physiological conditions, exhibit a reduction in HR activity via transcriptional or translational regulation of HR-associated factors. Thus, these combination strategies could limit cancer cell growth in response to PARPi and improve patient survival.

**Figure 8:**
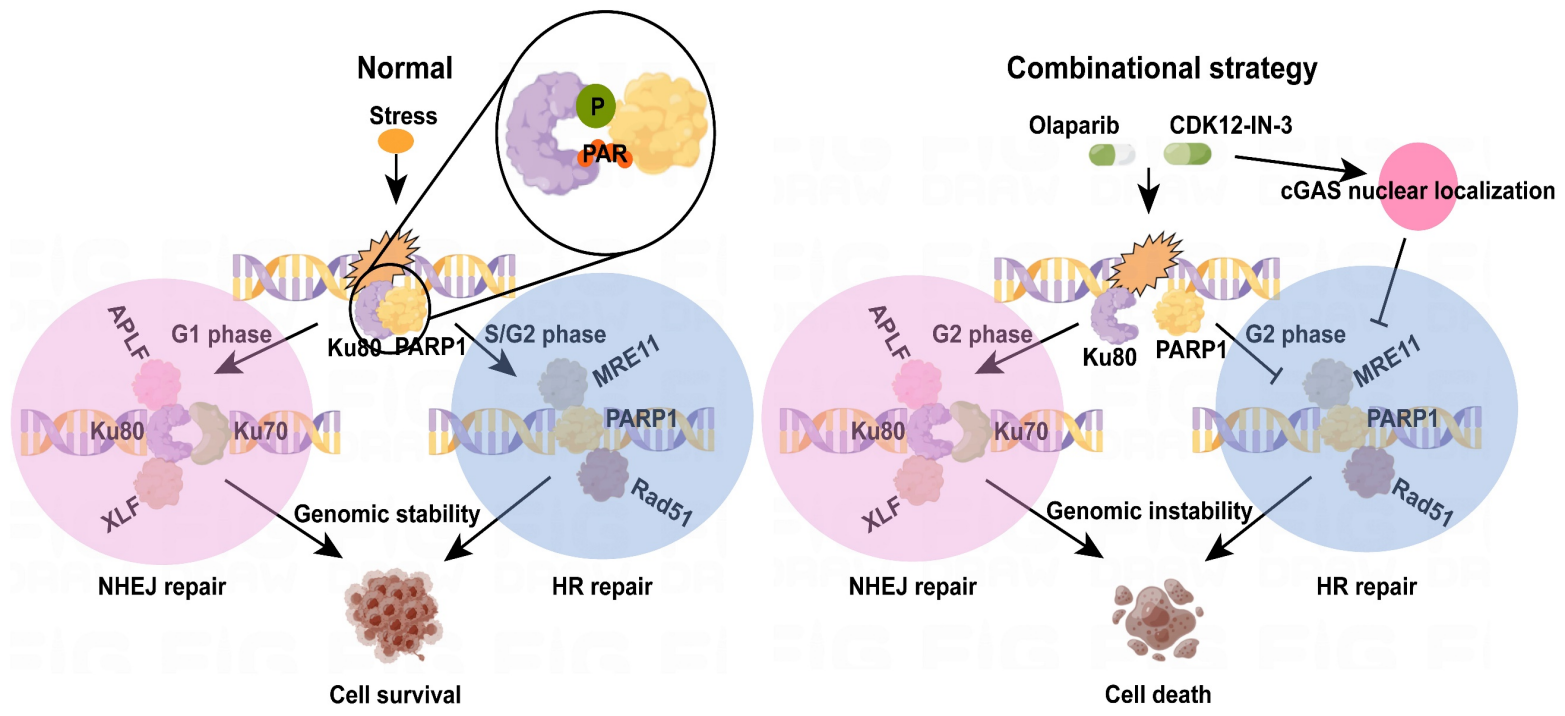
The diagram of mechanism of combination strategy.

Excessive activation of NHEJ pathway, and ensuing suppression of HR in G2 phase jointly undermine genome integrity. In this study, a novel CDK12 inhibitor, CDK12-IN-3, when cooperates with Olaparib, leads to fierce growth retardation of ovarian cancer cells *in vitro* and *in vivo*. Mechanically, posttranslational modifications of Ku80, phosphorylation and PARylation, were suppressed, which resulted in dissociation of the PARP1-Ku80 complex and further increased Ku80-mediated classical NHEJ (c-NHEJ) activity in the G2 phase as well as attenuation of HR activity, as detectable through evaluation of MRE11 foci and Rad51 foci. Intriguingly, cGAS nuclear relocalization under the combination treatment also suppressed HR activity. Thus, genomic instability triggered by the combination strategy ultimately caused cell death (Fig. 8).

Recently, accumulating evidence has suggested that posttranslational modifications are coupled to and thus affect the DDR, potentially regulating the biological processes of the DDR 43. The phosphorylation of HTATSF1, which is catalyzed by CK2, facilitates the formation of the HTATSF1-TOPBP1 complex in the S phase, which further promotes RPA/RAD51-dependent HR through the recognition of PARylated RPA by HTATSF1 44. In contrast to a posttranslational modifications cascade, ADP-Ribosylation and Phosphorylation on the same target, Histone H2B, control the adipogenesis. In detail, ADP-ribosylation of histone H2B-Glu35 suppresses AMPK-mediated phosphorylation of adjacent H2B-Ser36, thus, impedes the differentiation of adipocyte precursors^45^. We uncovered the necessity of both the PARylation and the phosphorylation of Ku80, not the MARylation, in maintaining the stability of PARP1-Ku80 complex for the first time. Though, to search exact sites, and whether and how these modification sites interplay with each other need to be investigated in future. As this complex is indispensable for the initial response of DDR, our research demonstrates the destruction of this complex would enhance Ku80-mediated NHEJ in DSBs, which disequilibrates the choice of repair pathway in G2 phase, when PARP1 acts as the pivotal scaffold or depositor in recruiting HR-associated factors^27^, ^46^, ^47^.

Though 53BP1 promotes NHEJ pathway while prevents HR repair through end protection, which cripples PARPi efficacy in BRCA1-deficient cells^6^. We cannot perceive significant changes over 53BP1 foci no matter in any single-agent group or combinational treatment group. Thus, it readily infers that chromatin-bound Ku complex might antagonize HR pathway, not the 53BP1 under combinational therapy. Kinetics experiments and biochemical techniques depict Ku complex compromises PARP1 and the MRN complex recruitment to damaged chromatin, of which the c-NHEJ is predominant, while the alternative NHEJ repair is intercepted^48^. Nevertheless, alternative end joining (alt-EJ) functions as an auxiliary DSBs repair pathway after end resection, also known as microhomology-mediated end joining (MMEJ), activated by MRN-CtIP complex^49^, it is originally thought that alt-EJ was enhanced as our data revealed HR activity was reduced. However, MRE11 foci is decreased under combinational treatment, indicative of weaken alt-EJ. In summary, it proposes a regulation model after combinational treatment that disassembly of PARP1-Ku80 at damaged chromatin heightens Ku80-mediated c-NHEJ, which further suppresses both HR and back-up alt-EJ through end protection in G2 phase. Altogether, genomic stability is encountered catastrophe in a couple of days, especially after DNA replication and cell division.

In addition to the innate immune response, dominated by cGAS-cGAMP-STING signaling, several evidence demonstrates cGAS is involved in genomic stability regulation^50^. After the insult of genotoxic agents (etoposide, camptothecin and H2O2), the nuclear cGAS-PARP1 interaction competes with PARP1-Timeless complex to inhibits HR repair, thereby induces severe DNA damage^33^. Consistent with this, in ovarian cancer cells, we verified that relocalization of nuclear cGAS suppressed HR activity after combinational treatment, while cGAS knockdown partially compromised DNA damage. However, cGAS-PARP1 interaction was not to reappear, possibly due to various genotoxic agents or cell type-specific. It is perplexing that cGAS decelerates replication forks in a manner of DNA-binding, which alleviates replication stress. On the contrary, cGAS-deficient cancer cells are more sensitive to radiation and chemotherapy^51^. Another report indicates that accumulation of cytosolic ssDNA, the product of the degradation of stalled replication fork, activates both the cGAS-STING and the P-body-dependent innate immune response^52^. Whether cGAS acts as a friend or foe? We presume that the subcellular localization, and the phase in genomic integrity maintenance, such as obstacle production, persistence, and elimination, might be the breakthrough. Indeed, appreciation of these intricate network is crucial for exploiting and applying anti-cancer drugs.

In addition, PARylation and phosphorylation of cGAS controls cGAS-mediated antiviral immunity and cGAS distribution^53^. Thus, how the suppression of the phosphorylation of cGAS Y215 under the combinational therapy is merit to explore in future. Intriguingly, recently, a remarkable study uncovered MRE11 nuclease-mediated mitochondrial DNA (mtDNA) degradation hyperactivates cGAS-STING-induced immune response^54^. It is readily reminiscent of whether MRE11-cGAS or cGAS-MRE11 cascade is also existed in nucleus, as the majority proportion of two molecules are in the nucleus after some chemical insults. Accordingly, the mechanism by which cGAS-inflicted HR suppression need to be further investigated, most likely interferes with HR initiation period-related factors or recombination period-associated factors.

Phosphorylation and PARylation are cornerstones in the interplay of cell cycle and DNA damage response, wherein relative targets have been exploited for anti-cancer therapy or overcoming chemoresistance^55^, ^56^. The Cyclin A2-CDK1 complex is characteristically formed in the S and G2 phase, which has been confirmed to interact with MRN complex^57^. After recruitment to damaged chromatin, NBS1 Ser432 is phosphorylated by CDK2, whilst CDK2 inhibition increases sensitivity to ionizing radiation^57^. When it turns to MRE11 subunit, CDK2 binds the Mre11 C-terminus, and this interaction is fundamental for CtIP phosphorylation and the consequent CtIP-BRCA1 formation, thereby, controls the HR capacity^37^. Consistent with this, we note that both cyclin A2 and CDK2 are diminished after combinational treatment, one is responsible for the G2 phase arrest, another explanation might be for the MRE11 foci reduction and HR suppression, suggesting multiple mechanisms to influence DNA damage response. Significantly, p21 prevents CDK-mediated phosphorylation of BRCA2 S3291, thus destabilizes RAD51-BRCA2 complex and decreases replication-coupled HR capacity, which is independent of its negative role in cell cycle progression^58^.

Here, we first utilized a highly efficient CDK12 inhibitor (CDK12-IN-3) in a combination approach that resulted in genomic instability by hyperactivating Ku80-mediated c-NHEJ while suppressing HR activity in the G2 phase in a phosphorylation- and PARylation-dependent manner. Through this combination strategy, HR-proficient ovarian cancer cells are resensitized to Olaparib, indicating that this combination is an evolutionarily stable therapy for patients with HR-proficient ovarian cancer and has more universal clinical value.

## Materials and Methods

### Cell culture and reagents

The ovarian cancer cell line SKOV3 was cultured in McCoy's 5A medium (Procell, PM150710), OVCAR3 cells were cultured in RPMI 1640 medium (Gibco), and the HEK293T cell line was cultured in DMEM (Gibco). All media were supplemented with 10% fetal bovine serum (Gibco) and 1% penicillin/streptomycin. DMSO (HY-Y0320), Olaparib (HY-10162), CDK12-IN-3 (HY-112261), CDK7-IN-1, and CCCP (HY-100941) were purchased from MedChemExpress. MMS (E0609) was purchased from Selleck.

### CETSA

SKOV3 cells treated with vehicle control (DMSO) or CDK12-IN-3 were analyzed via a CETSA^59^. The cells were cultured to 70-80% confluence and were then treated with either DMSO (vehicle control) or CDK12-IN-3 (50 nM) for 2 h. Following treatment, the cells were washed twice with PBS and distributed into ten PCR tubes (1 x 10^6 cells/tube in 100 μL of PBS). The tubes were incubated at various temperatures (37, 41, 44, 47, 50, 53, 56, 59, 63, and 67 °C) for 3 min in a thermal cycler, followed by 2 min at room temperature. The tubes were snap-frozen in liquid nitrogen, the cells were lysed via a freeze‒thaw cycle, and the soluble and insoluble fractions were separated via centrifugation at 12,000 RPM for 30 min at 4 °C. Western blotting was used to measure the expression of the target protein and internal reference protein after heating at various temperatures to obtain the curve showing the relationship between the parameter “Protein X/Loading control%” and the temperature, i.e., the melt curve for the CETSA, in the treatment group and the control group.

### Cell viability assay and Combination index (CI)

2×10^3^ to 5×10^3^ cells per well were seeded into 96-well plates, added with 100μl of fresh medium. After 24h, cells were treated with indicated drugs for 4 or 5 replicates. For designed days after treatment, cell viability was detected by the cell counting kit-8 assay according to the manufacturer (Vazyme Biotech, A311-01). The combination index was obtained applying data in cell viability using CompuSyn software.

### Colony formation assay

A total of 1500 cells were seeded per well in 6-well plates. After 24 h of incubation, the cells were treated with the indicated drugs for 12-14 days (3 replicates). The drug-containing medium was replaced every 2-3 days. Finally, the cells were fixed with 4% paraformaldehyde and stained with crystal violet. The colony number was determined via ImageJ software.

### EdU incorporation assay

A total of 1×105 cells per well were plated into 96-well plates, with 3 replicates per group. After being incubated overnight, the cells were treated with drugs for 48 h, and EdU incorporation was then assessed according to the instructions of the EdU Kit (RiboBio, C10310-1).

### Flow cytometry

Cells were cultured to 30-40% confluence in 6-well plates, with triplicate samples in each group. The cells were treated with the indicated drugs for 2-3 days. For apoptosis analysis, cells were detached and stained with the components of an Annexin V-FITC/PI apoptosis kit (BD, Catalog No: 556547). For cell cycle analysis, cells were first fixed with 75% precooled ethanol and then incubated at -20 °C overnight. Finally, the cells were washed with precooled PBS and stained with propidium iodide (PI) solution (BD, Catalog No: 556463). All these samples were analyzed via flow cytometry (BD).

### Organoid preparation, frozen sections and immunofluorescence

The protocols for the organoid-associated assays were described in our previous study^60^.

### Immunoblotting

After various treatments, cells were lysed in radioimmunoprecipitation assay (RIPA) buffer containing protease inhibitors and phosphatase inhibitors (Beyotime), and total protein was quantified via the bicinchoninic acid (BCA) method (Beyotime, P0010). Finally, the proteins were denatured, stored at -80 °C or used for immunoblotting analysis in accordance with the methods detailed in a previous study^61^. The primary and secondary antibodies used are described in Supplementary Table S1.

### Immunofluorescence (IF) assay

After different treatments, cells were fixed with 4% paraformaldehyde for 30 min, permeabilized in 0.5% Triton X-100 for 20 min, and blocked in 5% bovine serum albumin (BSA) for 30 min. Then, the cells were incubated with the indicated primary antibodies (Table S1) overnight. After that, the samples were washed three times with 0.05% PBST and incubated with the appropriate secondary antibody (Table S1) for 1 h. Finally, DAPI was added to stain nuclei (Invitrogen, P36931). A Zeiss confocal microscope was used to obtain images.

### Co-immunoprecipitation (Co-IP)

A total of 1×10^7 cells were washed three times with cold PBS before being lysed with 700 μl of IP lysis buffer (Beyotime, P0013) and then centrifuged. The cell lysates were immunoprecipitated with IgG or 1-4 μg of an IP antibody (Table S1) overnight at 4 °C. Then, to enrich the protein‒antibody complexes, 40 μL of protein A/G magnetic beads (Invitrogen, 80104G) was added for incubation for 2 h at 4 °C. The complexes were washed three times with PBST before boiling for 10 min.

### *In vitro* pull-down and coomassie blue staining

Recombinant 6×His-PARP1 protein (2 μg/per sample), purchased from Sino Biological (cat: 11040-H08B), was incubated with HEK293T cell lysates transfected with FLAG-Ku80 truncation plasmids overnight. Then, the complexes were incubated with His tag-conjugated beads for 2 h at 4 °C under constant rotation and washed three times before boiling for 10 min. For Coomassie blue staining, the reagent was purchased from Beyotime (P0017F), and the protocol was obtained from the manufacturer.

### Chromatin fraction assay

The method used for chromatin fractionation was described in a previous study^62^.

### BLRR system assay

The BLRR system-associated plasmids were purchased from Addgene (catalog no. 158958). The protocol for the related assay was described in previous research^22^. Five replicates were established for each treatment condition.

### siRNA transfection and plasmid transfection

Small interfering RNA (siRNAs) and plasmids were transfected into cells via Lipo8000^TM^ (Beyotime, C0533) according to the manufacturer's instructions. The sequence (5' to 3') of the siRNA targeting cGAS was: GGAAGAAAUUAACGACAUU. The plasmids were synthesized by Generalbiol (Chuzhou, China).

### Alkaline comet assay

A comet assay kit was purchased from Abcam (ab238544). After treatment with the indicated drugs for 48 h, cells were prepared for the assay according to the kit instructions. A Zeiss fluorescence microscope was used to acquire images. CASP software was used to analyze the tail moment.

### Senescence assay

After treatment with the indicated drugs for 48 h or recovery for 48 h, we used a Senescence β-Galactosidase Staining Kit (Beyotime, C0602) to examine the senescence status of the cancer cells. The detailed steps are described in the manufacturer's instructions. Then, we imaged the samples via microscopy.

### Xenograft model

The animal studies were conformed to the institutional ethics guidelines for animal experiments approved by the animal management committee of Nanjing Medical University (Approval no. IACUC-2207012). 3×10^6^ cells were injected into the one side axilla of the BALB/C nude mice (4 -week-old). After 1 week, when tumor was established newly, mice were randomized into four groups, comprised of DMSO group, CDK12i group, Olaparib group, and Combination group. Then, mice were treated with DMSO, CDK12i (0.5mg/kg), Olaparib (50mg/kg), or their combination once a day through intragastric administration for 14 days. Tumors were harvested at the same time point after 14 days of continuous treatment, and tumor size was measured every three days during this period. The formula for calculating tumor volume (V) is: V = (W² × L) / 2, where W represents tumor width and L represents tumor length. After euthanized, tumors, livers, and kidneys were used for further experiments.

### Immunohistochemistry (IHC) and haematoxylin and eosin (HE) staining

Tissues harvested from the mice were fixed with a 4% paraformaldehyde solution. The tissues were sequentially embedded, sectioned, deparaffinized, hydrated, subjected to antigen retrieval, blocked with serum, incubated with primary antibodies at 4 °C overnight and incubated with horseradish peroxidase-conjugated secondary antibodies. For HE staining, slides were stained with Mayer's hematoxylin (Sigma‒Aldrich) and counterstained with eosin Y solution (Sigma‒Aldrich). The primary and secondary antibodies used are listed in Table S1.

### Statistical analysis

Unpaired Student's t-test and One-way ANOVA were applied to examine differences between groups by GraphPad Prism 8.0.2. Significantly, p-values less than 0.05 were considered as statistically significant. Besides, *p* < 0.05: *, *p* < 0.01: **, *p* < 0.001: ***, *p* < 0.0001: ****.

## Supplementary Material

Supplementary figures and table.
