# Supplementary material for: Olaparib combined with CDK12-IN-3 to promote genomic instability and cell death in ovarian cancer
Source: Int J Biol Sci. 2024 Aug 19;20(11):4513–31. doi: 10.7150/ijbs.94568 (PMC11380446; doi:10.7150/ijbs.94568)
Supplement: Supplementary file 1 — Supplementary figures and table. [file ijbsv20p4513s1.pdf]

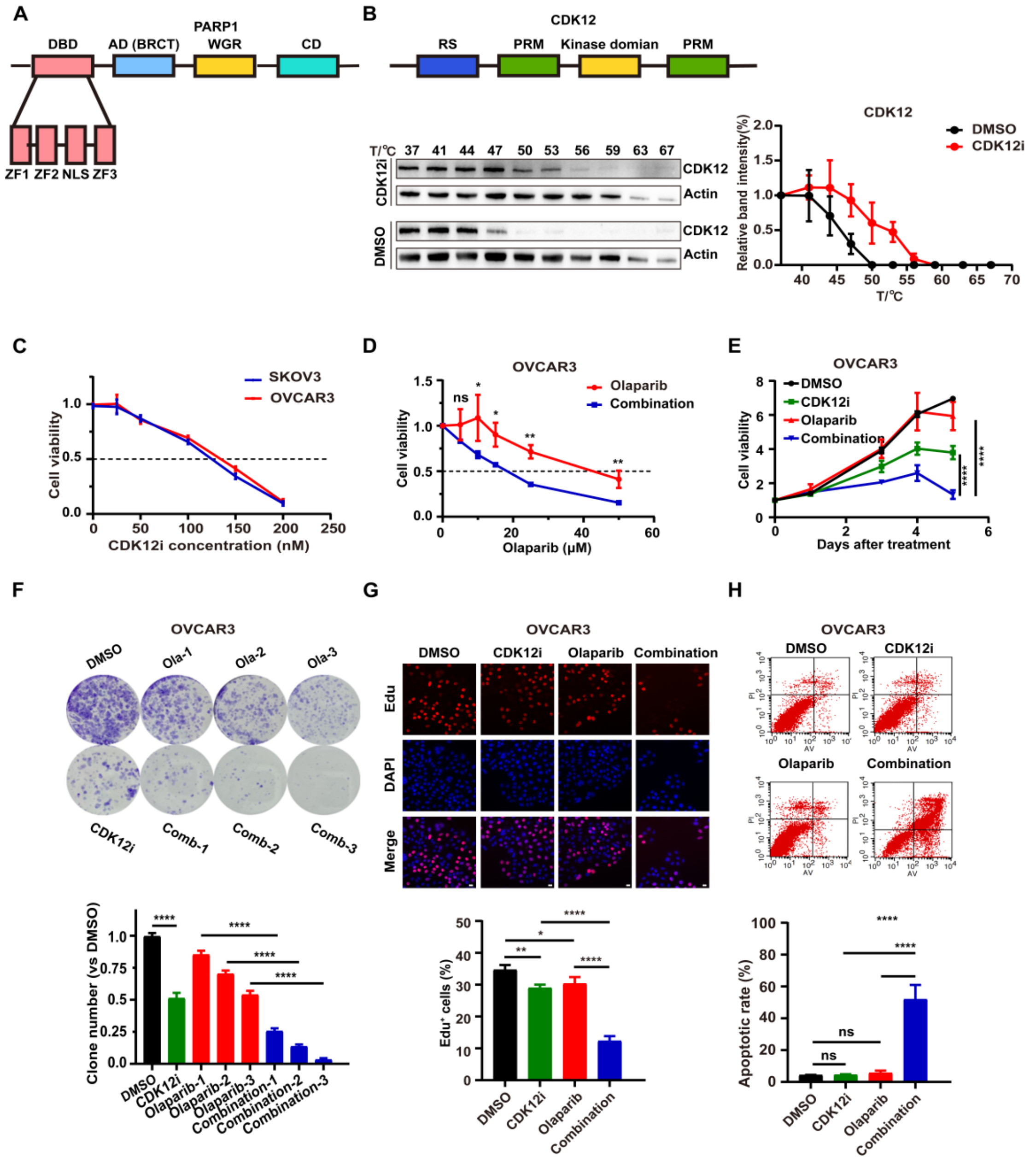

Supplementary Figure. S1 The synergistic sickness of Olaparib and CDK12-IN-3 in HR-proficient ovarian cancer cell. (A) The models about domains of PARP1. (B) The models about domains of CDK12 and Cellular thermal shift assay (CETSA, CDK12i: 50 nM, SKOV3 cells, at 37, 41, 44, 47, 50, 53, 56, 59, 63 and 67 °C). (C) IC50 of CDK12i in SKOV3 and OVCAR3. n = 3 independent experiments. (D-E) CCK-8 assay evaluated the cell viability of OVCAR3 cells after different treatments in a concentration- (CDK12i: 50nM, Olaparib: 0, 5, 10, 15, 25, 50 μM) and time- (CDK12i: 50 nM, Olaparib: 10 μM) dependent manner. n = 3 independent experiments. (F) Colony formation assay of OVCAR3 cells with different treatments. CDK12i: 15 nM, Ola-1: 0.5 μM, Ola-2: 1 μM, Ola-3: 2 μM. (G) Edu assay assessed the proliferation ability of SKOV3 cells (CDK12i: 50 nM, Olaparib: 10 μM, 2 days), scale bar: 20 μm. n = 3 independent experiments. (H) Apoptosis detection of SKOV3 cells under 48-72 h of different treatments by flow cytometry (CDK12i: 50 nM, Olaparib: 10 μM). n = 3 independent experiments.

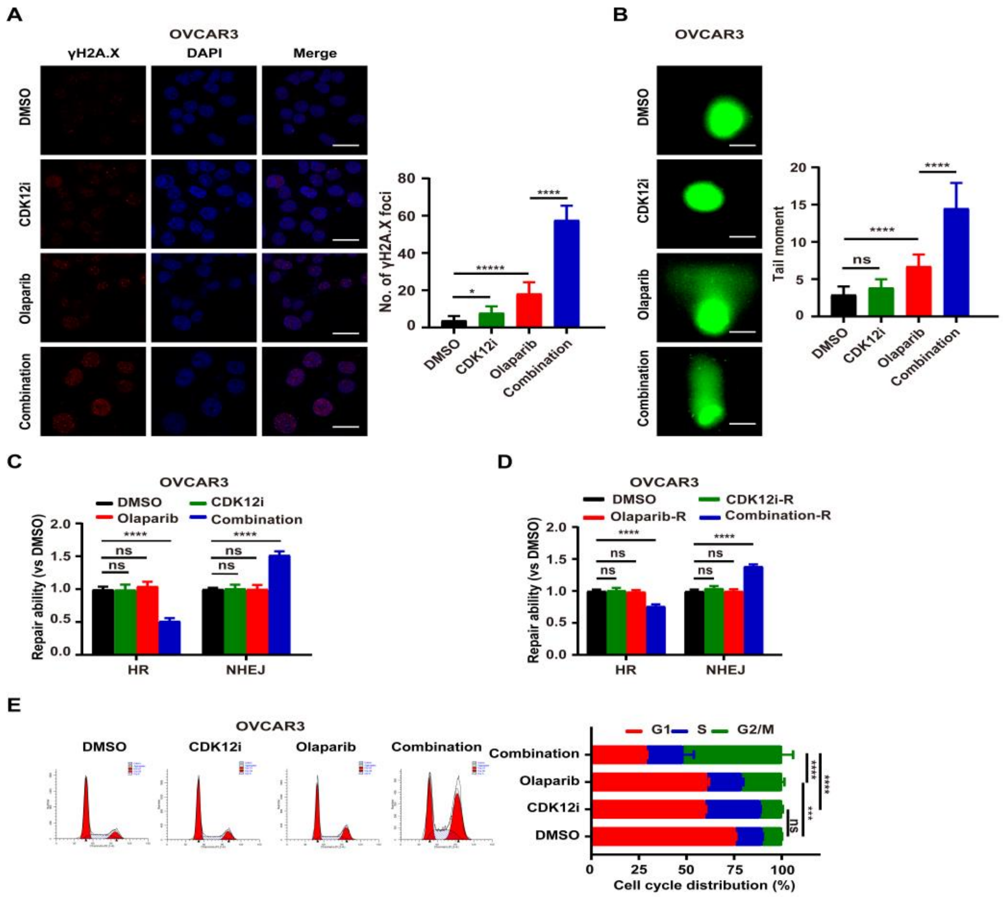

Supplementary Figure. S2 Combination of agents primes severe genomic instability and G2/M phase arrest in ovarian cancer cells. (A) γH2A.X foci staining via IF to visualize DSBs of OVCAR3 cells treated with drugs for 48 h (CDK12i: 50 nM, Olaparib: 10 μM), scale bar: 20 μm. (B) Comet assay showed changes in genomic stability of OVCAR3 cells under treatments for 48 h (CDK12i: 50 nM, Olaparib: 10 μM), scale bar: 10 μm. (C) BLRR system demonstrated NHEJ activity and HR activity of OVCAR3 cells after 48h treatment (CDK12i: 50 nM, Olaparib: 10 μM). n = 3 independent experiments. (D) After 48h treatment, OVCAR3 cells were cultured 48 h with fresh and drug-free medium, then NHEJ activity and HR activity of OVCAR3 cells were tested by BLRR system. n = 3 independent experiments. (E) Cell cycle distribution of OVCAR3 cells treated with drugs for 48 h-72 h by flow cytometry (CDK12i: 50 nM, Olaparib: 10 μM). n = 3 independent experiments.

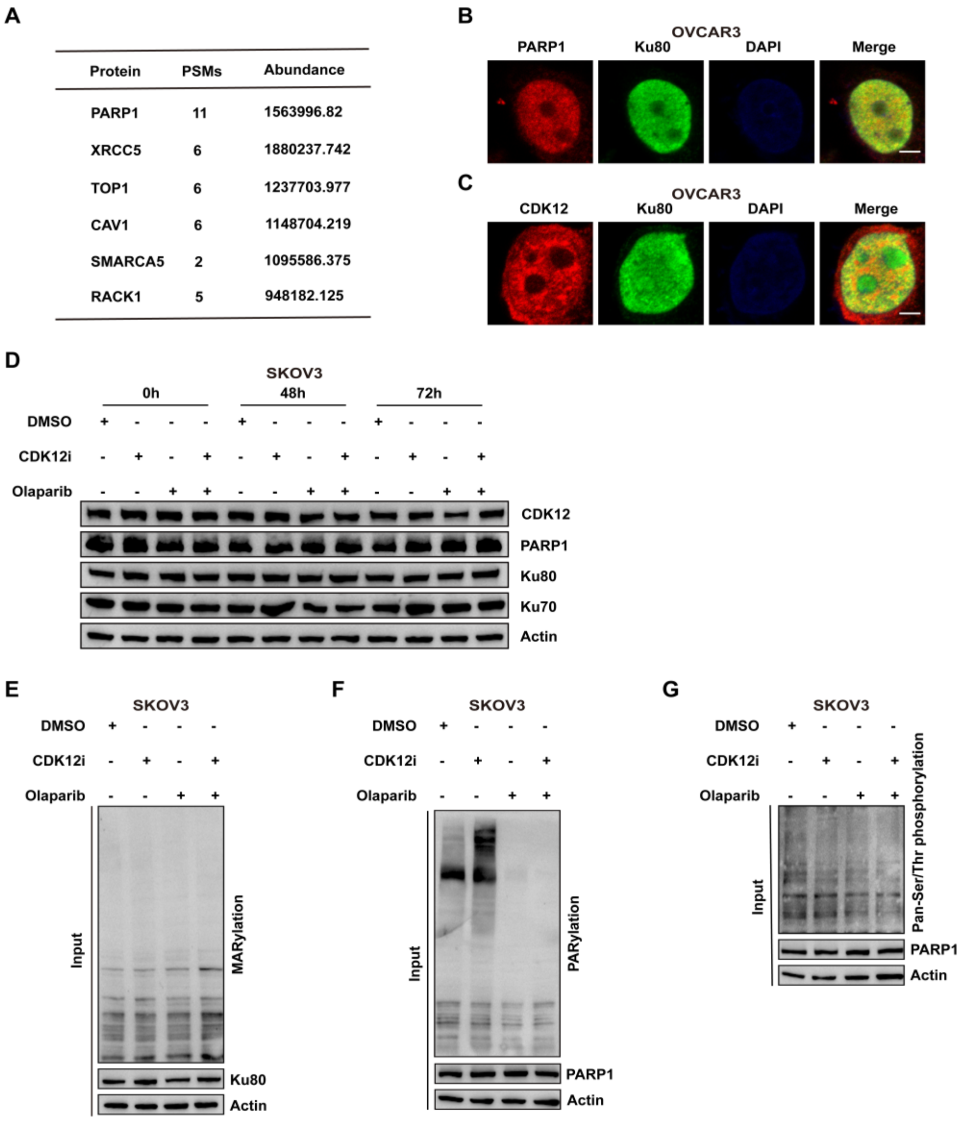

Supplementary Figure. S3 Ku80 acts as the co-target of Olaparib and CDK12i. (A) Partial MS data about IP-PARP1. (B) IF assays to visualize the co-localization of PARP1 and Ku80 in OVCAR3 cells, scale bar: 5 μm. (C) IF assays to confirm the co-localization of CDK12 and Ku80 in OVCAR3 cells, scale bar: 5 μm. (D) Total protein detection to evaluate the expression of CDK12, PARP1, Ku80, and Ku70 in SKOV3 cells after drugs treatment for 48 h and 72 h (CDK12i: 50 nM, Olaparib: 10 μM). (E-G) The total protein level examination, corresponding to Figure. 3G-3I.

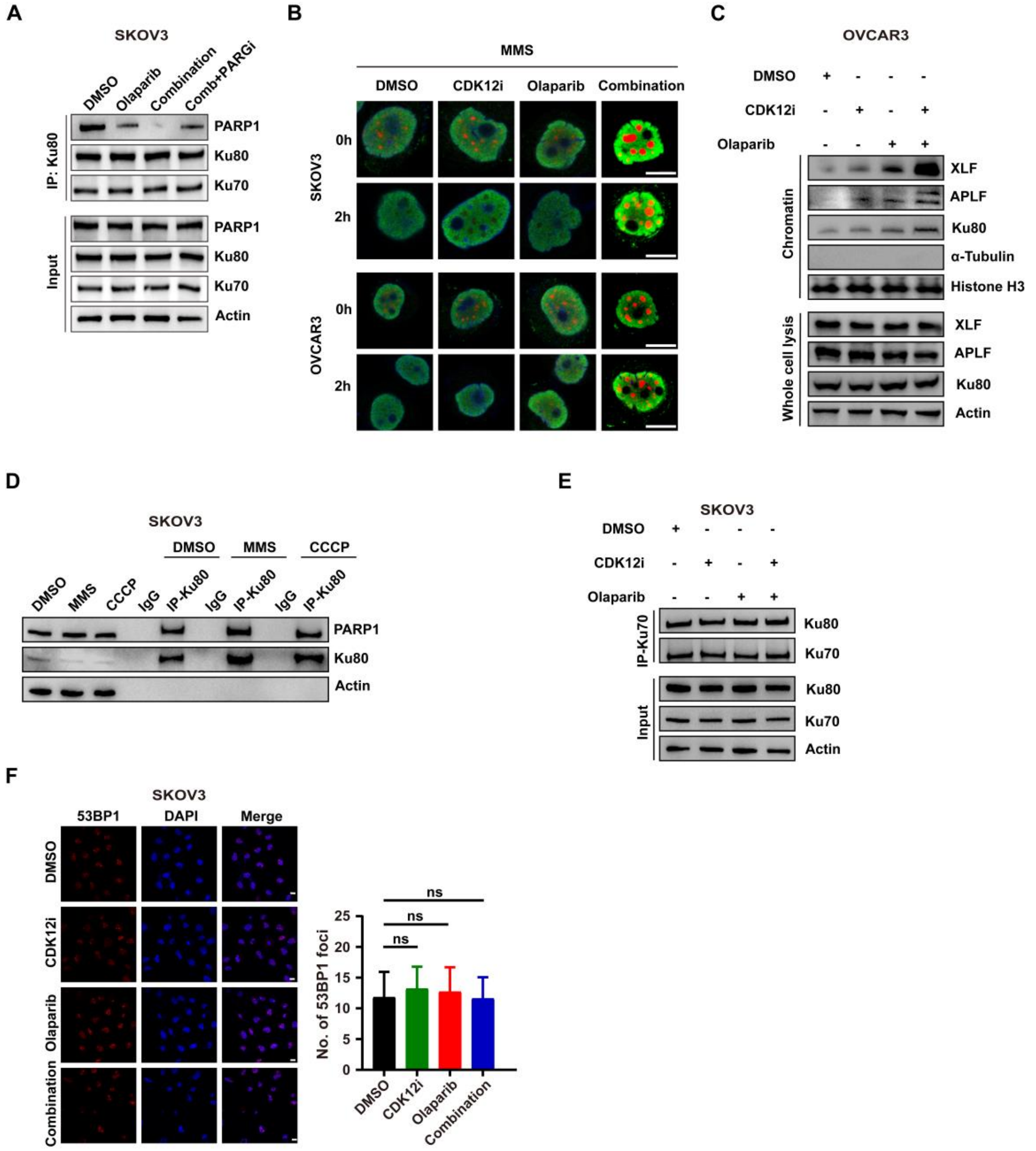

Supplementary Figure. S4 The combination therapy untimely activates NHEJ via disassembling the modifications-dependent PARP1-Ku80 complex. (A) Ku80 Co-IP followed by IB to investigate the PARP1-Ku80 complex under different treatments in SKOV3 cells (CDK12i: 50 nM, Olaparib: 10 mM, PARGi (PDD00031705): 15 μM, 2days). (B) After 48 h drugs treatment, SKOV3 and OVCAR3 were treated with 0.01% MMS for 1 h, then co-stained Ku80 and γH2A.X (CDK12i: 50 nM, Olaparib: 10 μM, 2 days), scale bar: 10 μm. (C) Chromatin fraction assay was used to confirm the enhanced Ku80-meidated NHEJ in OVCAR3 cells (CDK12i: 50 nM, Olaparib: 10 μM, 2 days). (D) Under normal status, OVCAR3 cells were treated with DMSO for 1 h, 0.01% MMS for 1 h, or 80 μM CCCP for 2 h, then the PARP1 Co-IP was conducted to detect the stability of PARP1-Ku80 complex. (E) PARP1 Co-IP to investigate the stability of Ku70/80 complex after different treatments in SKOV3 cells (CDK12i: 50 nM, Olaparib: 10 μM, 2 days). (F) IF assay to reveal the 53BP1 foci under different treatments in SKOV3 cells (CDK12i: 50 nM, Olaparib: 10 μM, 2 days), scale bar: 10 μm.

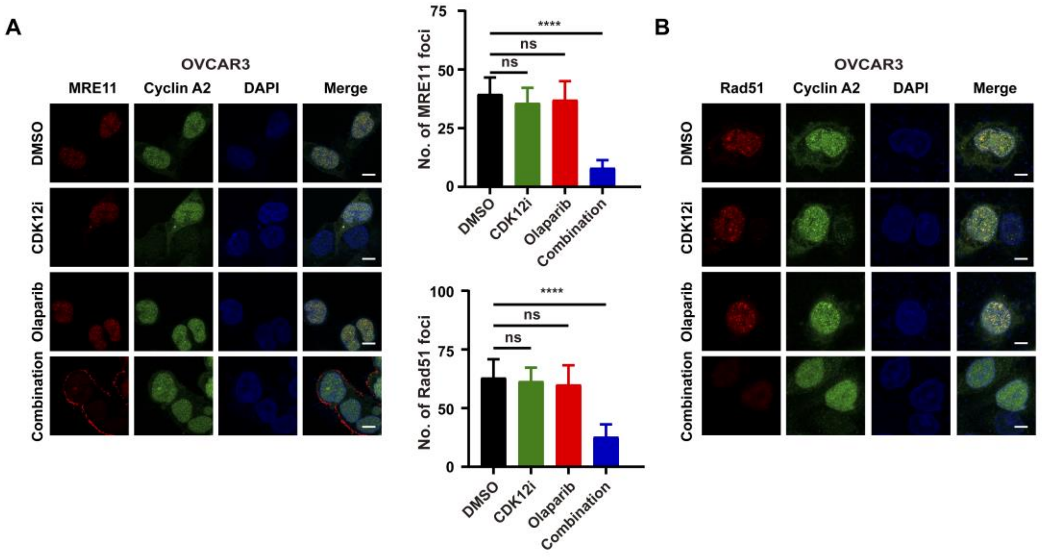

Supplementary Figure. S5 cGAS nuclear re-localization under combination treatment inhibits HR activity. (A-B) Visualization of MRE11 foci and Rad51 foci via IF assay in OVCAR3 cells with different treatments for 48 h followed by a transient 0.01% MMS treatment (CDK12i: 50 nM, Olaparib: 10 μM, 2 days), scale bar: 5 μm.

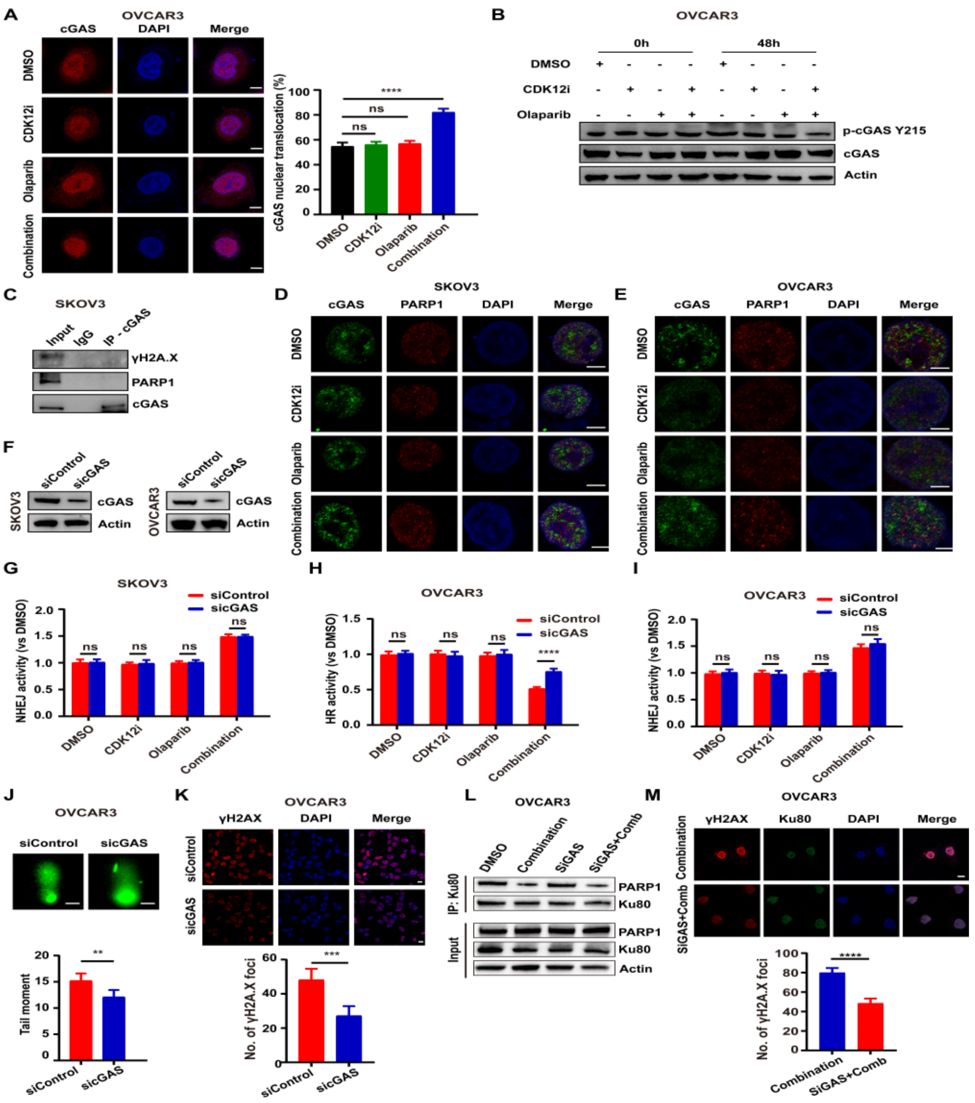

Supplementary Figure. S6 cGAS nuclear re-localization under combination treatment inhibits HR activity. (A) IF assay was applied to visualize the cGAS distribution in OVCAR3 cells (CDK12i: 50 nM, Olaparib: 10 μM, 2 days), scale bar: 5 μm. (B) cGAS Y215 phosphorylation in OVCAR3 was detected by IB using specific antibody (CDK12i: 50 nM, Olaparib: 10 μM, 2 days). (C) Co-IP assay to investigate whether PARP1 was the interactor of cGAS in SKOV3 cells. (D-E) IF assay to investigate whether cGAS and PARP1 co-localized in SKOV3 and OVCAR3, respectively (CDK12i: 50 nM, Olaparib: 10 μM, 2 days), scale bar: 5 μm. (F) Detection of the efficiency of sicGAS by IB. (G) BLRR system was applied to evaluate the role of cGAS in HR in OVCAR3 cells (CDK12i: 50 nM, Olaparib: 10 μM, 2 days). n = 3 independent experiments. (H-I) BLRR system was applied to evaluate the role of cGAS in NHEJ activity in SKOV3 and OVCAR3 cells, respectively (CDK12i: 50 nM, Olaparib: 10 μM, 2 days). n = 3 independent experiments. (J-K) Comet assay (scale bar: 10 μm) and IF assay (scale bar: 20 μm) to detect the role of cGAS contributing to genomic stability in OVCAR3 cells (CDK12i: 50 nM, Olaparib: 10 μM, 2 days). (L) Ku80 Co-IP to investigate the stability of PARP1-Ku80 complex after different treatments in SKOV3 cells (CDK12i: 50 nM, Olaparib: 10 μM, 2 days). (M) IF assay (scale bar: 10 μm) to examine the role of cGAS in genomic stability and Ku80 expression in OVCAR3 cells (CDK12i: 50 nM, Olaparib: 10 μM, 2 days).

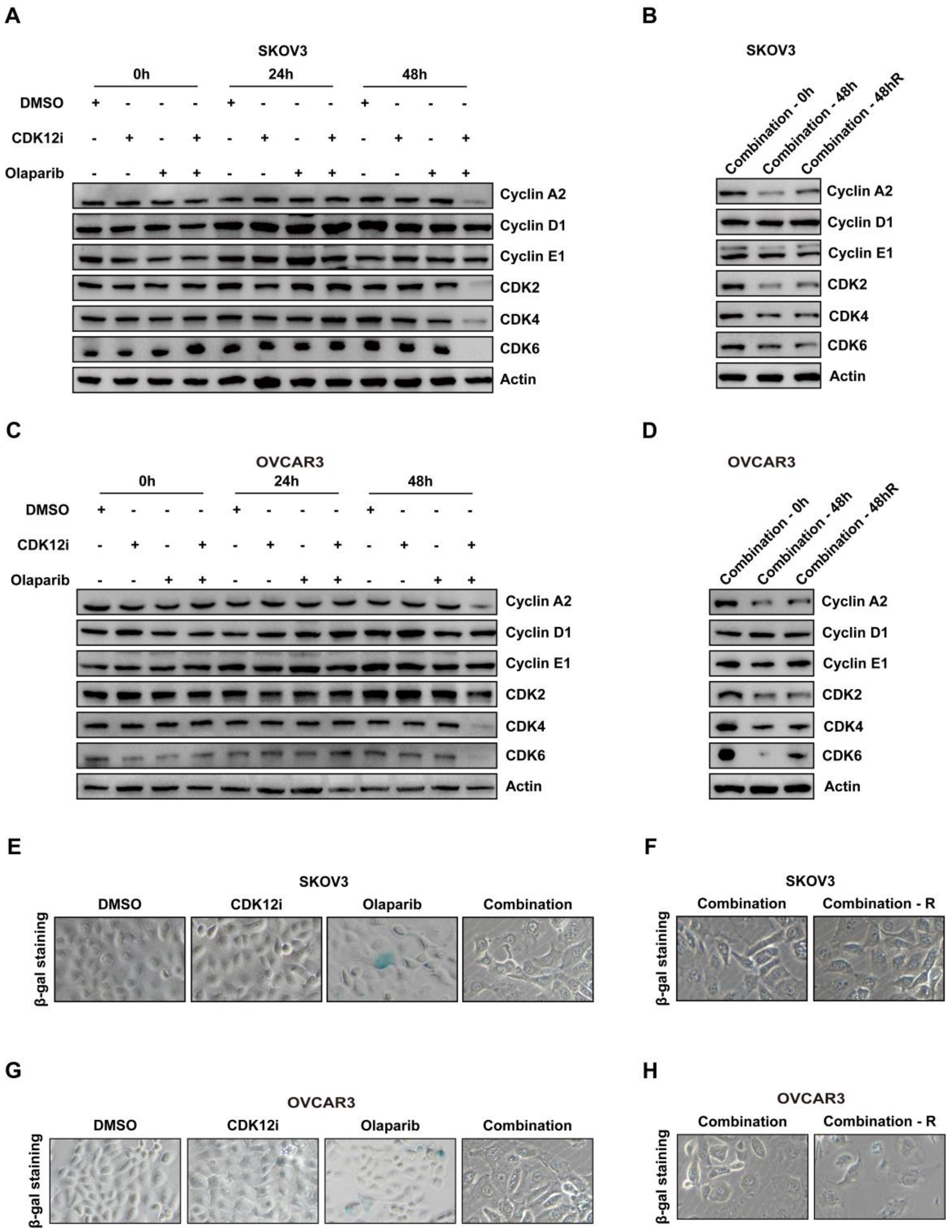

Supplementary Figure. S7 The patterns of several biomarkers reflecting cell fate. (A-B) cell cycle-related markers in SKOV3 cells. (C-D) cell cycle-related markers in OVCAR3 cells. (E-F) senescence status of SKOV3 cells by β-gal staining (CDK12i: 50 nM, Olaparib: 10 μM, 2 days treatment, 2 days recovery). (G-H) senescence status of SKOV3 and OVACR cells by β-gal staining (CDK12i: 50 nM, Olaparib: 10 μM, 2 days treatment, 2 days recovery).

Supplementary Table S1 Antibodies used in this study.

Table S1. Antibodies used in this study

Company	Antibody	Cat
Proteintech	XRCC5 Monoclonal antibody	66546-1-Ig
Proteintech	PARP1 Polyclonal antibody	13371-1-AP
Proteintech	CDK12/CRKRS Polyclonal antibody	26816-1-AP
Proteintech	MRE11 Polyclonal antibody	10744-1-AP
Proteintech	RAD51 Monoclonal antibody	67024-1-Ig
Proteintech	Histone H2A.X S139ph antibody	39118
Proteintech	Beta Actin Monoclonal antibody	66009-1-Ig
Proteintech	Histone-H3 Polyclonal antibody	17168-1-AP
Proteintech	Alpha Tubulin Polyclonal antibody	11224-1-AP
Proteintech	Annexin V Monoclonal antibody	66245-1-Ig
CST	CDK12 Antibody	11973
Merck	PARylation	MABE1031
Merck	MARylation	MABE1076
ABclonal	Ku80 Rabbit pAb	A5862
ABclonal	Ku70 Rabbit pAb	A7330
ABclonal	Phospho-CGAS-Y215 Antibody kit	RK05796
ABclonal	XLF Rabbit pAb	A19957
ABclonal	APLF Rabbit pAb	A17842
ABclonal	PanPhospho-Serine/Threonine Mouse mAb	AP1067
ABclonal	LC3B Rabbit mAb	A19665
ABclonal	SQSTM1/p62 Rabbit mAb	A19700
ABclonal	Bcl-2 Rabbit pAb	A0208
ABclonal	Cyclin A2 Rabbit mAb	A19036
ABclonal	Cyclin D1 Rabbit mAb	A19038
ABclonal	Cyclin E1 Rabbit pAb	A14225
ABclonal	CDK2 Rabbit pAb	A0294
ABclonal	CDK4 Rabbit pAb	A0366
ABclonal	CDK6 Rabbit pAb	A1545
ABclonal	Ki67 Rabbit mAb	A20018
ABclonal	DDDDK-Tag Rabbit mAb	AE063
Beyotime	Anti-Flag beads	P2115
Abcam	Goat Anti-Mouse IgG H&L (HRP)	ab205719
Abcam	Goat Anti-Rabbit IgG H&L (HRP)	ab205718
Abcam	Goat Anti-Rabbit IgG H&L (Alexa Fluor® 594)	ab150080
Abcam	Goat Anti-Rabbit IgG H&L (Alexa Fluor® 488)	ab150077
Abcam	Goat Anti-Mouse IgG H&L (Alexa Fluor® 488)	ab150113
Abcam	Goat Anti-Mouse IgG H&L (Alexa Fluor® 594)	ab150116
